# Elucidation of a non-thermal mechanism for DNA/RNA fragmentation and protein degradation when using Lyse-It

**DOI:** 10.1371/journal.pone.0225475

**Published:** 2019-12-02

**Authors:** Tonya M. Santaus, Ken Greenberg, Prabhdeep Suri, Chris D. Geddes

**Affiliations:** 1 Chemistry and Biochemistry Department, University of Maryland, Baltimore County, Baltimore, Maryland, United States of America; 2 Institute of Fluorescence, University of Maryland, Baltimore County, Baltimore, Maryland, United States of America; Meharry Medical College, UNITED STATES

## Abstract

Rapid sample preparation is one of the leading bottlenecks to low-cost and efficient sample component detection. To overcome this setback, a technology known as Lyse-It has been developed to rapidly (less than 60 seconds) lyse Gram-positive and–negative bacteria alike, while simultaneously fragmenting DNA/RNA and proteins into tunable sizes. This technology has been used with a variety of organisms, but the underlying mechanism behind how the technology actually works to fragment DNA/RNA and proteins has *hitherto* been studied. It is generally understood how temperature affects cellular lysing, but for DNA/RNA and protein degradation, the temperature and amount of energy introduced by microwave irradiation of the sample, cannot explain the degradation of the biomolecules to the extent that was being observed. Thus, an investigation into the microwave generation of reactive oxygen species, in particular singlet oxygen, hydroxyl radicals, and superoxide anion radicals, was undertaken. Herein, we probe one aspect, the generation of reactive oxygen species (ROS), which is thought to contribute to a non-thermal mechanism behind biomolecule fragmentation with the Lyse-It technology. By utilizing off/on (Photoinduced electron transfer) PET fluorescent-based probes highly specific for reactive oxygen species, it was found that as oxygen concentration in the sample and/or microwave irradiation power increases, more reactive oxygen species are generated and ultimately, more oxidation and biomolecule fragmentation occurs within the microwave cavity.

## Introduction

Lyse-It is a revolutionary cellular lysing platform that can lyse bacteria, viruses, human, and plant cells alike in under 60 seconds[1–5]. In general, Lyse-It utilizes a standard 900W Frigidaire microwave with a special sample chamber equipped with equilateral gold triangles in the center. The gold triangles serve as microwave focusing antennas of which the theory and design[6] have been extensively reviewed and tested with a variety of bacteria including *Bacillus anthracis*, *Neisseria gonorrhea*, *Chlamydia trachomatis*, and most recently *Listeria monocytogenes* and *Vibrio cholerae*.[3, 5–13] At early lysing times (<30 seconds) on the platform, intact genetic material and biomolecules are released, allowing for proteomics and whole genome sequencing to be undertaken. Lysing occurs due to the rapid expansion of water which exceeds the mechanical strength of cell walls. At later times (>30 seconds), biomolecules, DNA/RNA, proteases, and nucleases[14] are degraded where the fragment size is a function of both time and/or microwave power (energy) applied to the lysing process. This power/time process allows for tunable base-pair sizes to be produced, simultaneously shredding the sample matrix. Subsequently, quantitative Polymerase Chain Reaction (qPCR) can be applied directly to sample lysates *without the need* for further sample preparations, which is a paradigm-shift in sample preparation today.

In this paper, we identify the generation of solution-based species which are believed to be responsible for DNA cutting and protein degradation using Lyse-It, i.e. reactive oxygen species (ROS). These reactive oxygen species, in particular singlet oxygen (^1^O_2_), superoxide anion radicals (O_2_^•-^), and hydroxyl radicals (•OH), are already implicated in oxidative damage of fatty acids, DNA, RNA, and proteins in unrelated studies.[15] Oxidation of proteins from ROS normally occurs upon reaction with specific functional groups of target proteins leading to protein modifications.[15] Fragmentation of the protein peptide chain, aggregation of crosslinked reaction products, enzyme inactivation, and accumulation of altered cellular protein products are also a few of the damages that can be caused by ROS.[16] When these ROS are over-produced, an imbalance occurs between the excessive ROS and limited antioxidant defenses causing oxidative stress.[15] Oxidative DNA products from ROS/oxidative stress include 8-hydroxy-deoxy guanosine, 8-hydroxy-adenine, and 7-methyl-8-hydroxy-guanine.[16] Along with oxidized DNA products, single and double strand breaks can arise, removal of nucleotides from a sequence, base modifications, and DNA-protein crosslinks can occur.[16] Additionally, ROS that surpass antioxidant defenses lead to breaks in fatty acid chains and increases in membrane fluidity and permeability of lipids. All of the above damages to proteins, DNA, and lipids lead to chronic diseases and disorders including mitochondrial diseases, Parkinson’s disease, aging, cancer, and diabetes.[16] Herein, we show an increase in the detection of ^1^O_2_, •OH, and O_2_^•-^ as microwave irradiation power and time increase with Lyse-It. Additionally, we demonstrate oxidative effects on Fe^2+^ and Fe^3+^ as a function of increasing microwave irradiation power and time, further confirming the generation of ROS. From this work, we show that these ROS contribute significantly to DNA and protein degradation.

## Materials and methods

### SGS PAGE of conventionally heated and microwave irradiated samples both *with* and *without* Lyse-It

1 ± 0.1 grams of ground beef or spinach was added to a 50 mL conical tube and suspended in 20 mL of DI water. The ground beef was broken down into even smaller pieces using a scoopula and the spinach was ground using a mortar and pestle prior to suspension. This aided in the release of red blood cells from the ground beef into the DI water and for spinach cells to be disseminated into solution. The suspended samples were then centrifuged at 14,000 rpm for 10 minutes to pellet large pieces of food product. The supernatant was used for conventional heating and microwave irradiation. 1 mL of ground beef supernatant was aliquoted into 1.5 mL microcentrifuge tubes for conventional heating or into isolators adhered to blank microscope slides or Lyse-It slides for microwave irradiation. Ground beef and ground spinach were conventionally heated in a Fisher Scientific Isotemp heating block for 1 minute at 10°C temperature intervals between 40°C and 80°C. Samples were also microwave irradiated for 60 seconds at 10, 20, 30, 40, and 50% power both *with* or *without* Lyse-It. It is important to note that 30% power refers to 30% power of a 900 W total power microwave cavity (i.e. 270W). Samples were cooled to room temperature prior to loading on a 10% SDS PAGE. 20 μL of cooled sample was loaded into the respective gel wells and were run at 150 V for approximately 45 minutes and then stained in Oriole overnight. The gels were rinsed in DI water to remove excess stain and imaged using a BioRad GelDoc EZ imager.

### Purging of ground spinach and ground beef with subsequent analysis on SDS PAGE

1 ± 0.1 grams of ground beef or spinach was added to a 15 mL conical tube and suspended in 15 mL of DI water then centrifuged at 14,000 for 10 minutes. 1250 μL of sample supernatant was added to a sample chamber on a blank microscope or Lyse-It slide (see www.Lyse-It.com). S1 Fig shows a Lyse-It slide and the Lyse-It purging experimental system. A sample lid was adhered to the sample chamber to create a sealed system. A venting needle was placed above the sample and the “gas-in” needle was placed in the sample. A continuous stream of bubbles was created and held constant for 10 minutes. After purging, the samples were microwave irradiated for 60 seconds at 30% power. Post microwave irradiation, samples were cooled to room temperature prior to loading on a 10% SDS PAGE. 20 μL of cooled sample was loaded into respective gel wells. The gels were run at 150 V for approximately 45 minutes and then stained in Oriole overnight. The gels were rinsed in DI water to remove excess stain and imaged using a BioRad GelDoc EZ imager.

### *V*. *cholerae*, *L*. *monocytogenes*, *and S*. *aureus* DNA fragmentation purging and lysing with Lyse-It for subsequent Agilent 2100 Bioanalyzer analysis

Bacterial suspensions (10^8^ CFU/mL) of *V*. *cholerae*, *L*. *monocytogenes*, or *S*. *aureus* were made following McFarland standards. Bacteria were then purged utilizing the same purging procedure as ground spinach and ground beef as described in the methods section above. Following purging, the sample was microwave irradiated at either 30% power (270 W) for 30 seconds, or 50% power (450 W) for 60 seconds. Samples were cooled to room temperature and the intracellular components and cells were pelleted using ethanol precipitation. Ethanol precipitation was performed where 500 μL of sample was mixed with 1000 μL cold ethanol. Two 1.5 mL microcentrifuge tubes were made of the same sample mixture so that the pelleted components could be concentrated into one tube. After mixing by pipetting, the samples were centrifuged at 14,000 rpm for 20 minutes. The supernatant was discarded, and the pellet was dried at room temperature for 10 minutes. 50 μL of DI water was added to the dried pellet to rehydrate and re-suspend the pellet in solution. For the 2100 Agilent Bioanalyzer preparation, all samples were diluted down by 50% (10 μL sample to 10 μL DI water). All samples were analyzed at the Institute of Marine and Environmental BioAnalytical Lab in Baltimore, MD. USA, on an Agilent 2100 Bioanalyzer using the *DNA high sensitivity kit* (Agilent).

### Absorption and fluorescence measurements of gas purged probe(s)

To investigate the effects of oxygen concentration on the absorption and fluorescence of the probe/ROS reaction, samples were purged with either argon (~0% O_2_), air (~16% O_2_), or oxygen (~100% O_2_) for 15 minutes in a sealed Lyse-It apparatus (i.e., same purging layout for bacteria as food products). Following purging, the sample was microwave irradiated at 30% power (270 W) for 60 seconds. Samples were cooled to room temperature (22°C ± 0.5°C). Once samples were cool, the sample lid was removed (as the reaction was complete) and fluorescence spectra were obtained on an in-house built optical train. The system is equipped with a LaserMate BML-473-nm laser and Ocean Optic HR2000+ spectrometer. Following fluorescence acquisition, an aliquot of the sample was measured using a Cary 60 UV-Visible spectrophotometer. This sequence was followed for the three probes and the three purging environments respectively.

### Absorption and fluorescence detection of probe/ROS reactions during microwave irradiation

Absorption and fluorescence spectra of the ROS probe were obtained *pre* and *post* microwave irradiation both *with* and *without* Lyse-It. Absorption and fluorescent spectra were obtained on the stock probe concentration solution and is considered the *Pre* (initial) sample. Fluorescence detection of the probe/ROS reaction was acquired on the optical train described in the above methods section. After obtaining *pre*-absorption and fluorescence spectra, 1 mL of the selected probe was microwave irradiated for 60 seconds at 10%, 30% and 50% power both *with* and *without* Lyse-It. Post microwave irradiation, samples were cooled to room temperature. Once the samples had cooled to room temperature, fluorescence spectra were obtained, followed by absorption spectra.

### pH and metal oxidation

pH and metal oxidation were studied to look at any potential pH change post microwave irradiation and to observe any oxidation of metal ions that could occur within the microwave cavity. pH of *pre* and *post* microwave irradiated (50% power, 60 seconds cooled, no Lyse-It) deionized water was measured with Sigma Aldrich pH Test Strips and with an Accumet Basic AB15 pH meter.

Oxidation studies were performed on 2.5 mM Iron (II) chloride (FeCl_2_), 2.5 mM, and 25 mM Iron (III) chloride (FeCl_3_). It is important to note that the Fe^2+^ solutions were a mixture of Fe^2+^ and Fe^3+^ as some of the Fe^2+^ was already oxidized into Fe^3+^; however, there were still quantities of Fe^2+^ still present. Additionally, the mechanism of oxidation for Fe^2+^ and Fe^3+^ is still under investigation as to which oxidative iron products were formed. 1 mL of each metal salt suspension was microwaved irradiated 10 → 50% power for 30 seconds or 60 seconds. After each microwave irradiation, absorption spectra were acquired. Photographs were taken after each set of microwave power ranges (10 → 50% power).

Additionally, 2.5 mM Iron (II) chloride was purged under argon, air, or oxygen for 10 minutes. Directly after purging, the sample was microwave irradiated with Lyse-It for 60 seconds at 30% power. Absorbance of the stock 2.5 mM Iron (II) chloride was obtained as well as cooled Iron (II) chloride post purging and microwave irradiation. The integrated absorbance was calculated between 200 and 400 nm. Statistical analysis of the integrated absorbance was not calculated as the integrated absorbance served as an additional validation of a bathochromic shift, and hence oxidation of Fe^2+^ and Fe^3+^.

## Results and discussion

### There is more ground beef and ground spinach protein extracted and subsequently degraded using Lyse-It

**Fig 1:**
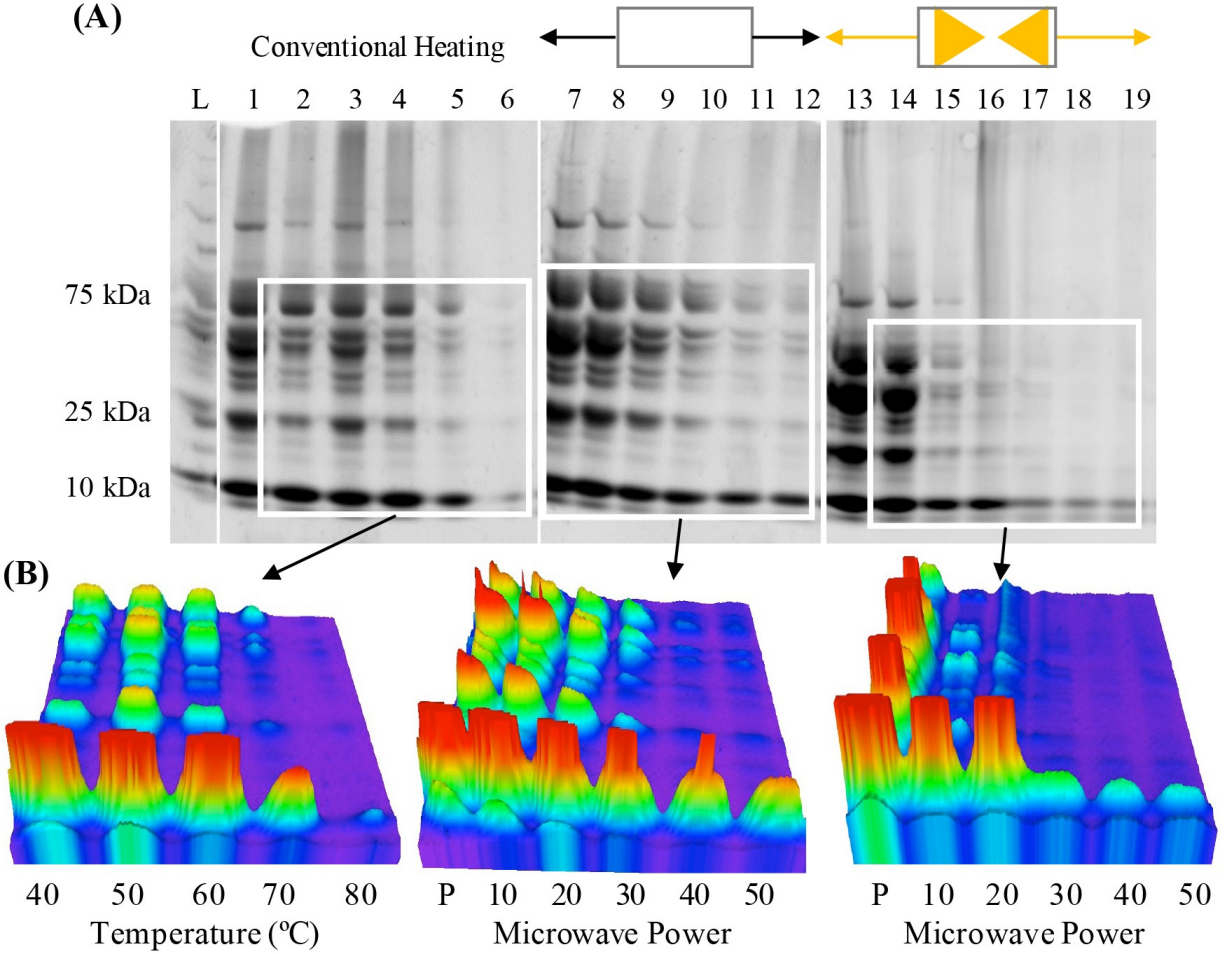
SDS PAGE of ground beef (A) and the 3D images (B) of conventional heating (left), no Lyse-It (middle) and with Lyse-It (right). More protein was extracted and subsequently degraded with Lyse-It as a function of increasing microwave power. L: kDa ladder, Lanes 1–6: pre, 40–80°C, Lanes 7–12: standard microwave irradiation (no Lyse-It) 10–50% power, 60 seconds, Lanes 13–19: microwaving with Lyse-It 10–50% power, 60 seconds.

One of the major advantages of Lyse-It is the technology’s ability to rapidly and efficiently, extract intracellular components from bacterial cells, while secondly fragmenting and degrading DNA and proteins at higher and longer microwave power and time settings. To explore this concept in more detail, ground beef and ground spinach were conventionally heated, or microwave irradiated both with or *without* Lyse-It. Protein extraction and subsequent degradation of ground beef and ground spinach was analyzed through SDS PAGE and 3D SDS PAGE, Fig 1 and S2 Fig respectively. In both cases, high conventional heating temperatures were required to degrade a wide variety of size proteins. For ground beef, significant amounts of protein were released with lower temperatures and substantial degradation was seen at > 70°C (Fig 1A **left**). When microwave irradiation both with or *without* Lyse-It was used, more ground beef protein was extracted as compared to *Pre* and low conventional heating temperatures. When ground beef was exposed to standard microwave irradiation (i.e. no Lyse-It), significant protein degradation was seen at 30% power (Fig 1A **middle**). On the contrary, protein degradation began to become evident with Lyse-It at only 20% power, 60 seconds, which is lower in overall microwave cavity energy as compared to standard microwave irradiation. (Fig 1A **right**). It can be seen that lower molecular weight proteins are less effected by microwave irradiation both *with* and *without* Lyse-It. We believe that larger proteins are affected by microwaves first and as those proteins get degraded, the microwaves take effect on lower molecular weight proteins along with fragments of previously degraded proteins. Protein degradation is more clearly evident in the 3D images for all conditions tested (Fig 1B). As conventional heating temperature increased or as microwave irradiation power increased, the intensity of the various protein peaks become less intense demonstrating that protein has been degraded. Interestingly, Lyse-It appears to release more protein quickly, which then rapidly degrades at both higher microwave powers and longer times.

Similarly, to ground beef, spinach displays comparable trends in protein release and subsequent degradation as shown by SDS PAGE and 3D SDS PAGE (S2 Fig). As conventional heating temperature increases, the degree of protein fragmentation increases (S2 Fig **left**). For standard microwave irradiation, protein is released in significant amounts at early powers, but as the power increases, the band intensity of the protein decreases indicative of degradation (S2A Fig **and middle**). Microwaves can also affect enzymes. Increasing microwave power and irradiation time decreases the enzyme activity while still allowing for DNA to be detected through quantitative Polymerase Chain Reaction. The degree to which enzymes are still active is also tunable using Lyse-It.[17]

The significant differences in the food product protein gels and the amount of protein both extracted and degraded between the three methods, subsequently led to the investigation of increasing the oxygen content in the sample, while keeping both microwave power and time constant. There clearly was a secondary degradation pathway underway, in addition to heating. These observations posed critical questions as to whether an additional non-thermal biomolecule degradation mechanism was also in play when using Lyse-It.

### Oxygen content and the degree to which sample temperature affects protein extraction and degradation

**Fig 2:**
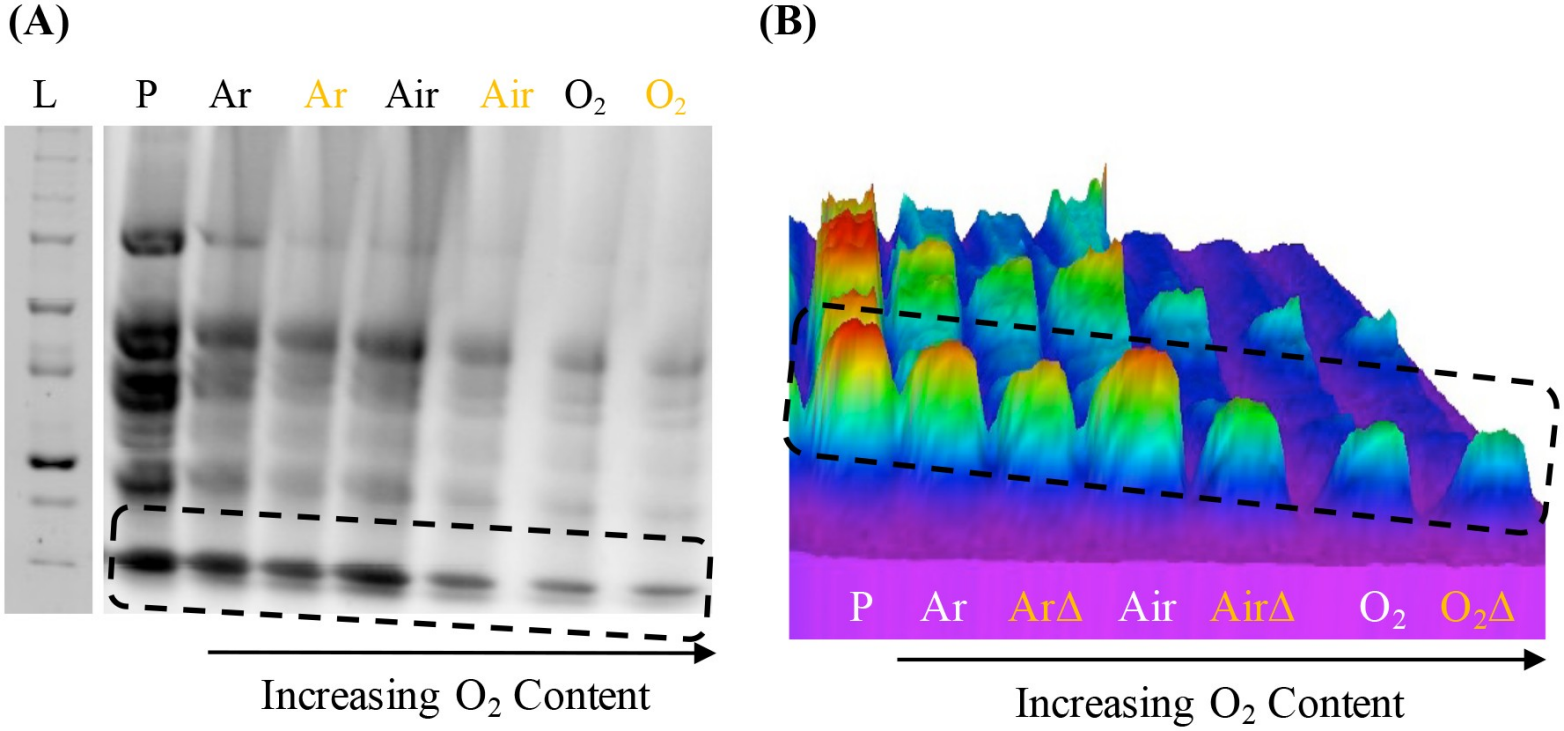
SDS PAGE (A) of purged ground beef with and without Lyse-It (30% power, 60 seconds) and the respective 3D plot (B). Protein extraction is seen with the Pre (P) ground food samples. Protein degradation is then seen as the oxygen content increases. ArΔ –Argon purged samples with Lyse-It; AirΔ –Air equilibrated samples with Lyse-It; O_2_Δ –Oxygen purged samples with Lyse-It.

It was shown in the last section that as microwave power or sample temperature increases, protein degradation also increases. Thus, it was important to look at possible additional factors including oxygen content and constant microwave power and time, thus, constant temperature, especially given that reactive oxygen species (ROS) are well known to degrade biomolecules.[16, 18, 19] Both ground beef and ground spinach were purged with either argon (~0% O_2_), air (~16% O_2_), or oxygen (~100% O_2_) and subsequently microwave irradiated both *with* or *without* Lyse-It at 30% power for 60 seconds. SDS PAGE of both food products—ground beef and spinach, was performed and can be seen in Fig 2 and S3 Fig respectively. The above microwave condition was selected because clear protein bands could be distinguished on the gels. The SDS PAGE and 3D SDS PAGE of ground beef are seen in Fig 2. It is clear in the 2D image that as oxygen content increases (i.e., argon to air to oxygen), the amount of protein degradation increases (i.e., fainter bands are observed as oxygen content increases–Fig 2A). The difference in the intensity of the peaks adds to this analysis as post microwave irradiation, the intensity of the peaks decreases from *Pre* and as oxygen concentration increases, the peaks become much less intense. Also, when comparing a purged gas microwave irradiated sample both *with* or *without* Lyse-It, the band intensity with Lyse-It is significantly less than that of the band without Lyse-It (Fig 2B). This is consistent with the previous food product gels where using Lyse-It increases the amount of protein degradation. Interestingly, bands with no oxygen, i.e. argon purged samples, are significantly greater than those with oxygen. This strongly suggests that an ROS mechanism is responsible for protein degradation.

To further study the hypothesis that oxygen content had an effect on protein degradation, the same purging and lysing experiments were performed on ground spinach. Ground spinach was purged and then lysed both *with* and *without* Lyse-It for 60 seconds at 30% power. For samples that were irradiated both *with* and *without* Lyse-It, as oxygen content increases, the number of distinguishable bands from *Pre* (i.e. the sample before lysing) became smeared (S3 Fig). The loss of band intensity, like that seen in the solid box with solid arrow and dashed box and arrow, as compared to the increase in smearing, is indicative of continued and increasing protein degradation.

**Fig 3:**
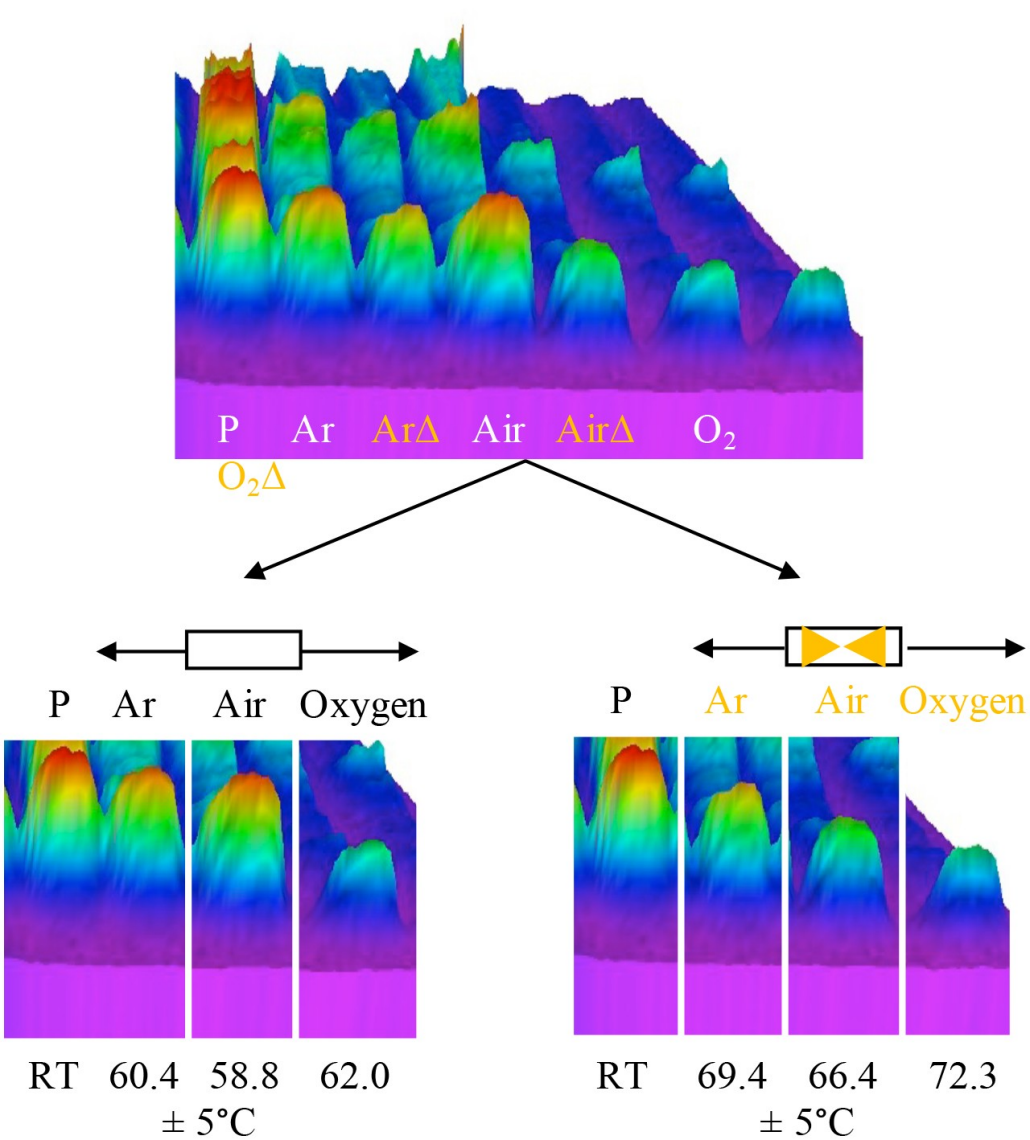
Break-down of the 3D ground beef purging SDS PAGE. Temperatures for the samples are shown below the gel and each temperature has an error of 5°C. As oxygen content increases, the overall temperature of the sample remains within error. Thus, another mechanism is additionally responsible for an increase in protein degradation. RT = Room temperature (21.0 ± 3°C). ArΔ –Argon purged samples with Lyse-It; AirΔ –Air equilibrated samples with Lyse-It; O_2_Δ –Oxygen purged samples with Lyse-It.

One of the pressing questions that was posed after analyzing the previous gels was what role temperature played (if any) in the differences in protein degradation. To understand the potential temperature role, the 3D SDS PAGE image of ground beef was *further analyzed* into its respective *with* and *without* Lyse-It purged components. Temperatures of each of the post microwave irradiations with an error of 5°C are shown below the respective purging gasses (Fig 3). For standard microwave irradiations (no Lyse-It), the temperatures of the irradiations where not significantly different, an average temperature of ~60.4°C. Concurrent with the standard no Lyse-It data, the temperatures with Lyse-It for each of the purged and lysed samples were not significantly different, an average of ~69.4°C. It is important to note, that the comparison of temperatures is *not* between standard microwave irradiation and Lyse-It. The temperature comparison analysis is key between the samples of the *same irradiation type* (i.e., no Lyse-It *or* Lyse-It within the same data set). Thus, it can clearly be seen that protein degradation is not just a function of temperature, but moreover another contributing non-thermal factor.

### Oxygen concentration effect on DNA fragmentation of three bacteria–*V*. *cholerae*, *L*. *monocytogenes*, and *S*. *aureus*

After the studies of protein extraction and degradation through conventional heating and microwave irradiation both *with* and *without* Lyse-It, it was important that DNA fragmentation also be investigated in varying oxygen concentrations. The three bacteria were prepared as discussed in the above methods section. Total (summed) DNA fragment concentration (pg/μL) was calculated for base pairs below 200, 500, 1500, 3000, 4500, 6000, 7500, 9000, and 10000 bp. Additionally, concentrations were summed between various base pair ranges (<200, 200–500, 1500–3000, 4500–6000, 6000–7500, 7500–9000, and 9000–10000 bp). Ranges slightly differed between bacteria. Any base pairs that were reported in the raw data of the Bioanalyzer below 35 bp and above 10000 bp were not used as those base pairs were the lower and upper markers of the Agilent High Sensitivity DNA kit. It is important to note that for the analysis, *total concentration of extracted DNA* between the different microwave power and times is key for the understanding of microwave power comparisons within a specific purging gas.

**Fig 4:**
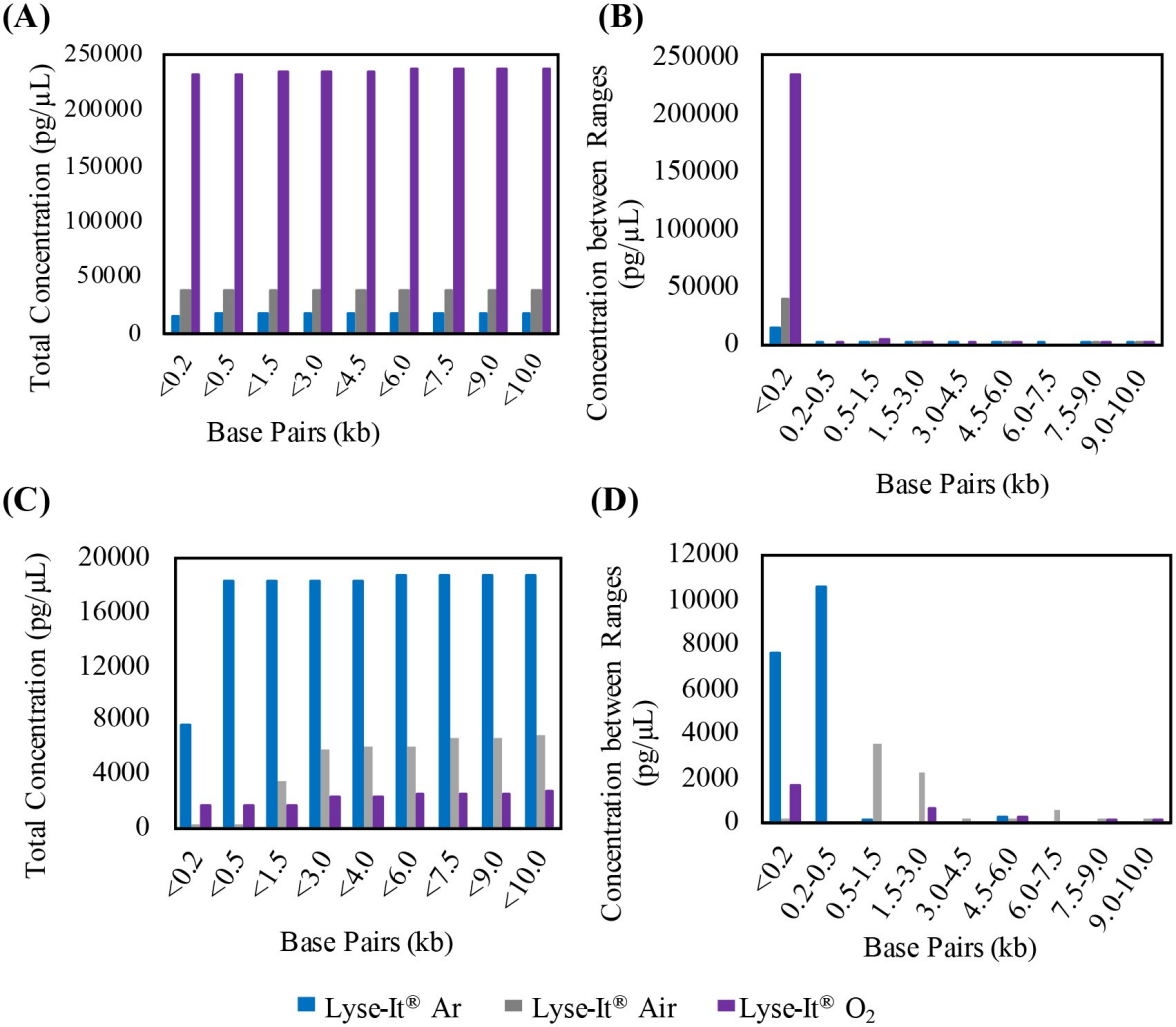
2100 high sensitivity DNA Bioanalyzer of *V*. *cholerae*. Bacteria were purged for 15 minutes and then lysed with Lyse-It for 30 seconds at 30% power. (**A and B)** and 50% power, 60 seconds **(C and D)** Total concentrations (pg/μL) below the reported base pairs post 30% power, 30 seconds (**A)** Concentrations (pg/μL) between base pair ranges for 30% power 30 seconds, **(B)** Total concentration before the reported base pairs post 50% power, 60 seconds **(C)**, and concentration between base pair ranges for 50% power, 60 seconds **(D)**.

For *V*. *cholerae* the amount of extracted DNA is orders of magnitude greater when the bacteria was lysed at 30% power 30 seconds (Fig 4A) with Lyse-It as compared to being lysed under much harsher conditions, i.e. for 50% power, 60 seconds (Fig 4C). This is consistent across argon, air, and oxygenated samples. It was expected that as the concentration of base pairs was summed, the total concentration of DNA would increase (Fig 4A and 4C). Additionally, when the concentrations were summed only between ranges, the highest concentration of DNA would be seen for the lower base pair sizes (i.e. 200 and 500 base pairs) (Fig 4B and 4D). Intuitively, it was assumed that as oxygen content increases, the total number of fragments and thus the total concentration would also increase. This trend *was indeed seen* with *V*. *cholerae* at 30% power for 30 seconds but was *inverse* at a 50% power, 60 seconds setting. At high energies such as 50% power for 60 seconds (27kJ) for Gram-negative bacteria like *V*. *cholerae*, the extracted DNA is significantly fragmented into a wide range of sizes, some of which can be both below and above the Bioanalyzer marker limits (35 and 10000 bp). Therefore, individual comparison of powers between purging gasses was utilized. At low power and shorter times (30% power, 30 seconds), the total DNA fragmentation for *V*. *cholerae* purged under oxygen and subsequently lysed with Lyse-It was ~236,000 pg/μL while at higher powers and longer times the concentration dropped to ~18,000 pg/μL. When purged under air at low power and time settings, the total concentration was almost 40,000 pg/μL and dropped to ~8,000 pg/μL at 50% power. Finally, under argon at low powers the concentration was ~18,000 pg/μL and ~3500 pg/μL at high power and longer time. The most significant drop in total DNA concentration was observed for oxygen purging where the total concentration dropped 13x that of the low power and short time setting (Fig 4A and 4C). For purging with air and oxygen, the DNA concentration dropped more than 5x.

This significant drop in DNA concentration following oxygen purging and subsequent lysing with Lyse-It suggests that in more oxygenated environments, there is more generation/ diffusion of reactive oxygen species to the sample, thereby possibly fragmenting more DNA. Within the *V*. *cholerae* study, it was also seen that smaller fragments with a higher total concentration are seen at lower power and time settings, i.e. 30%, 30 seconds (Fig 4B and 4D). Additionally, what can be observed from this data is that no matter what the purging gas, a wide variety of base pair sizes can be seen, and that at lower microwave power and irradiation times, more DNA can be extracted.

**Fig 5:**
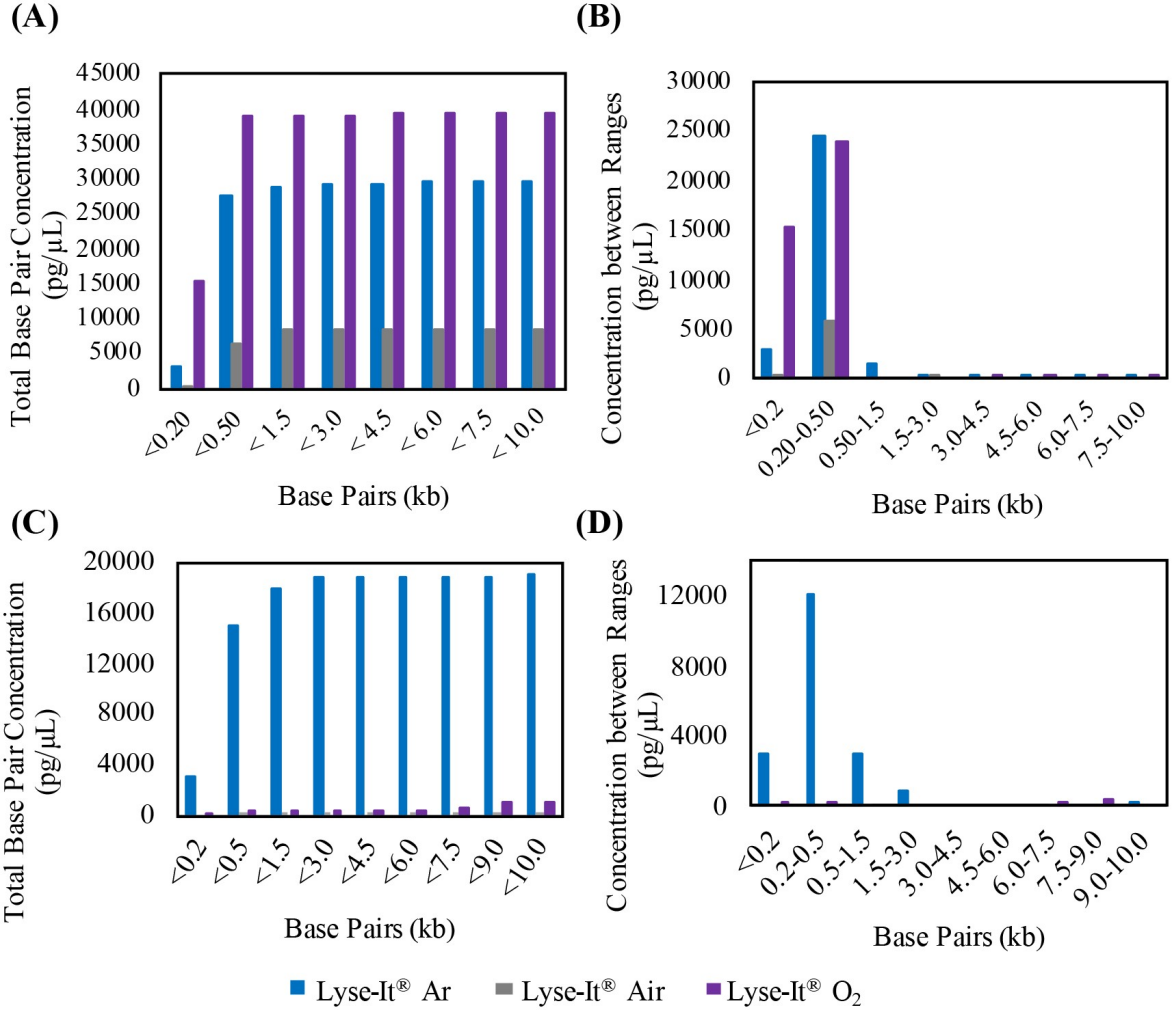
2100 high sensitivity DNA Bioanalyzer *S*. *aureus*. Bacteria were purged for 15 minutes and then lysed with Lyse-It for 30 seconds at 30% power **(A and B)** and 60 seconds at 50% power **(C and D)**. **A and C)** Total concentrations (pg/μL) below the reported base pairs. **B and D)** Concentrations (pg/μL) between base pair ranges.

For Gram-positive bacteria where cellular lysing requires more energy, i.e. higher microwave power and longer times, *L*. *monocytogenes* followed similar trends as discussed above for *V*. *cholerae* (Fig 5). For the highest oxygen concentration in the sample, at low power and short time, the total concentration was ~13,800 bp and at high power (50%), the concentration dropped to ~4,300 bp. This was a decrease of 3.2x the total concentration at low power. Further analysis showed an increase of 1.6x for air and a decrease of 3.2x for argon. What is very interesting about this set of data is that the drop in the total DNA concentration under an oxygenated environment is that it is almost equal to that in the drop for argon. We attribute this anomaly to a potential balance between the number of lysed cells, and therefore extracted DNA after purging with argon, to a significant amount of released cells and subsequent DNA fragmentation at high power. Even more interesting is the case where the total concentration increased under an air environment.

Though beyond the scope of this research, it is not out of the question that at both high and low powers, especially in air environments that *reactive nitrogen species* (RNS) may well play a role in DNA fragmentation as well as reactive oxygen species.

Finally, *S*. *aureus* was analyzed under the same conditions as *V*. *cholerae* and *L*. *monocytogenes* and just like the other two bacteria, the total concentration of fragmented DNA was greater at lower microwave power and shorter time settings (S4 Fig). For both oxygenated environments, there were significant drops in total DNA concentration post lysis. What was very interesting, in this case, is that the total concentration in an argon environment did not drastically change as compared to air and oxygen environments.

Following these studies of DNA fragmentation in varying oxygen environments, it was concluded that as oxygen content increases and the sample is microwave irradiated at low power and time, DNA is being fragmented more so than in the other gaseous environments. This leads us to believe that the role of reactive oxygen species is prevalent within the sample thus, the amount of DNA being fragmented is substantial. Under high microwave power and longer time conditions, the bulk of the extracted DNA is being significantly and continually broken down (fragmented) in oxygenated and in air environments.

To investigate *what* reactive oxygen species are *actually present* in microwave irradiated samples, fluorescent off/on probes were subsequently used for the specific detection of singlet oxygen (^1^O_2_), hydroxyl radicals (•OH) and superoxide anion radical (O_2_^•-^).

### Detection of ^1^O_2_, ·OH, and O_2_^·-^ through fluorescent probes SOSG, HPF, and DHE respectively

The commercially available fluorescent probes Dihydroethiudium (DHE) detects superoxide anion radical (O_2_^·-^), Singlet Oxygen Senor Green (SOSG) detects singlet oxygen (^1^O_2_) and hydroxyphenyl fluorescein (HPF) detects hydroxyl radicals (•OH). Similar to the fragmentation of DNA/ proteins at various oxygen sample concentration studies, each fluorescent probe was purged under argon (~0% O_2_), air (~16% O_2_), or oxygen (~100% O_2_), and then subsequently lysed at 50% power, 60 seconds with Lyse-It, to understand what ROS were being generated. *Pre* absorption and fluorescence values of the probes were taken when the probe was suspended in DI water and subjected only to the air environment. The *Pre* of each probe under argon and oxygen were also tested and it as found that the *Pre* spectra were not significantly different in the various environments. Thus, all microwave irradiations of probes were compared to both the *Pre* absorption and fluorescence spectra in air.

**Fig 6:**
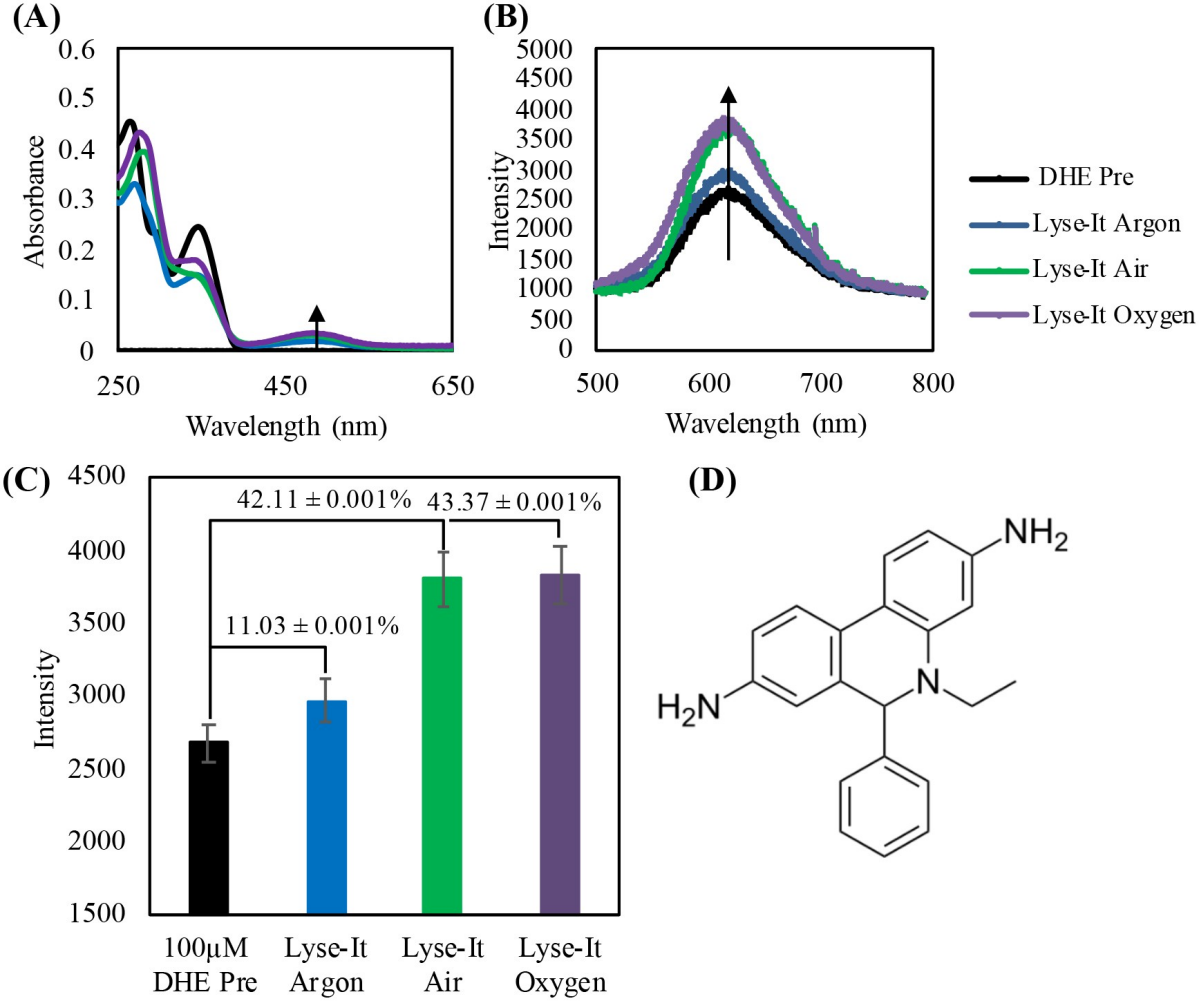
100 μM DHE purged then irradiated at 50% power, 60 seconds with Lyse-It **(A)** Absorption spectra of DHE pre and post microwaved irradiation, **(B)** Fluorescence spectra pre and post purging and microwave irradiation, **(C)** Fluorescence λ_max_ intensity with the percentage increase from *Pre* with respect to increasing oxygen concentration. **(D)** Dihydroethidium unreacted probe. As oxygen content increases, the peak at approximately 475-nm in the absorbance spectra and 605-nm in the fluorescence spectra increases indicating an increase in the detection of superoxide anion radical.

From the absorption spectra for DHE, when the probe reacted with very little to no O_2_^·-^, there is very low absorption between 400 and 600 nm. However, upon reaction with O_2_^·-^, DHE becomes hydroxylated yielding a 2-hydroxyethiudim product[20] that now absorbs between 400 and 600 nm.[20–22] 2-hydroethidium is a three ring conjugated system and thus a new peak becomes evident in the absorption spectra between 400 and 600 nm. The absorption of the product increases as more DHE is converted to its product and PET is quenched or ‘turned-off’ (Fig 6A). Subsequently, this product can then be excited at 473-nm to excite the fluorescent product and generate a fluorescent signature correlating to the reaction and subsequent detection of O_2_^·-^. An increase in the fluorescent signature of 2-hydroxyethidium as the oxygen concentration in the sealed sample increases, can be seen in Fig 6B. The percent increase relative to the unreacted DHE can be calculated under the assumption that there is a 5% error for each of the λ_max_ values. Relative error is then calculated from the high, original, and low λ_max_ for each sample (*lysed argon*, *lysed air*, *and lysed oxygen)* divided by the original *Pre* λ_max_. The percentage increase from the unreacted, original DHE probe can be seen in Fig 6C, where the most significant change in intensity was observed for both the air (16% O_2_) and oxygenated (~100% O_2_) environments. This detection of O_2_^•-^ confirmed that more O_2_^•-^ can be generated from microwave irradiation in oxygenated environments.

**Fig 7:**
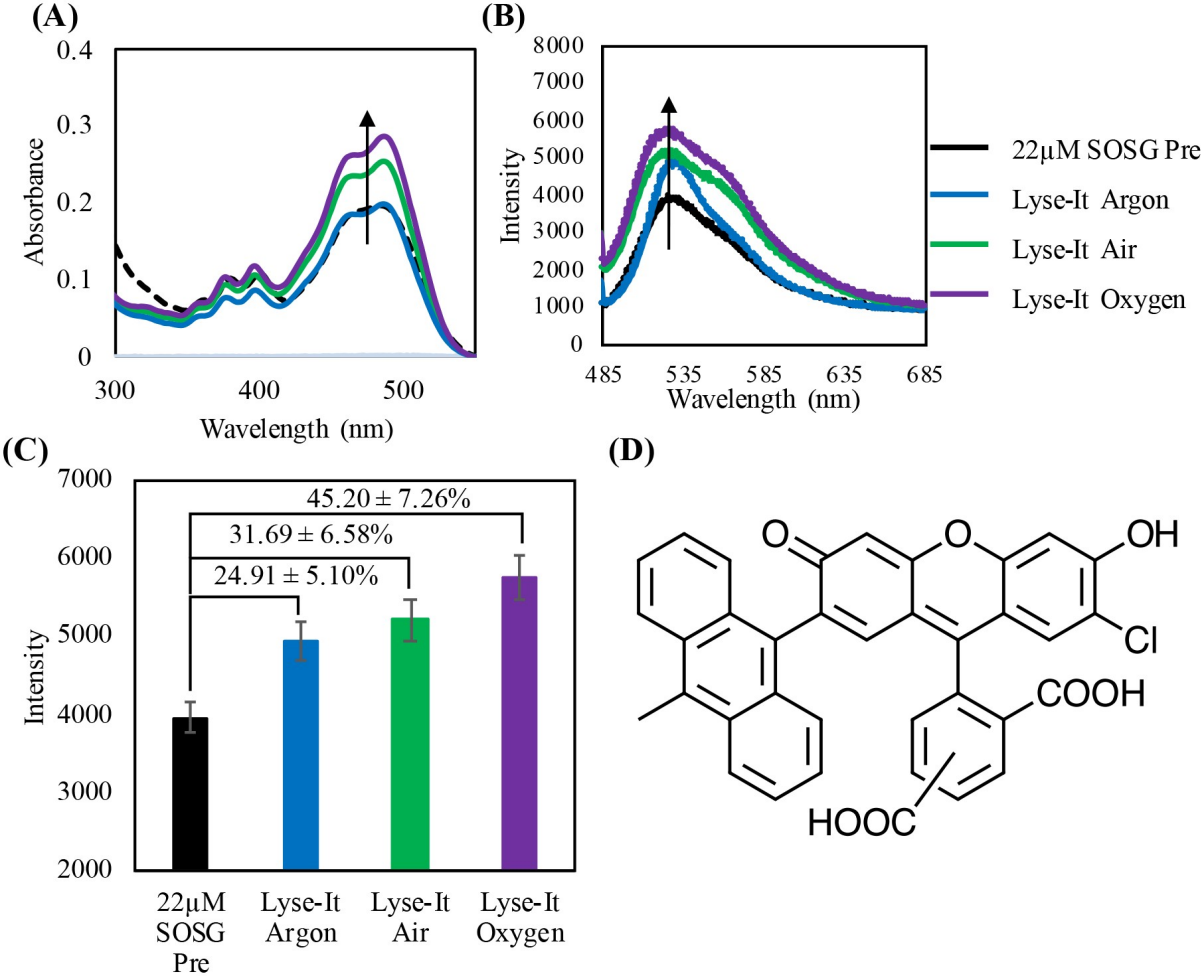
22 μM SOSG purged then irradiated at 30% power, 60 seconds with Lyse-It **(A)** Absorption spectra of SOSG pre and post microwaved irradiation, **(B)** Fluorescence spectra pre and post purging and microwave irradiation, **(C)** Fluorescence λ_max_ intensity with the percentage increase from *Pre* with respect to increasing oxygen concentration. **(D)** Singlet Oxygen Sensor Green unreacted probe structure. As oxygen content increases, the peak at approximately 475-nm in the absorbance spectra and 530-nm in the fluorescence spectra increases indicating an increase in the detection of singlet oxygen.

Additionally, 22 μM SOSG was suspended in DI water, purged, and then microwave irradiated with Lyse-It at 50% power, 60 seconds. Just like DHE, a 5% error was assumed for each of the λ_max_ fluorescent values. As the oxygen concentration in the sample increased, the fluorescent intensity also increased (Fig 7B). The percent increase from *Pre* SOSG was calculated and shown in Fig 7C. In oxygenated environments, more singlet oxygen is being generated and the percentage significantly increases from the *Pre* value.

**Fig 8:**
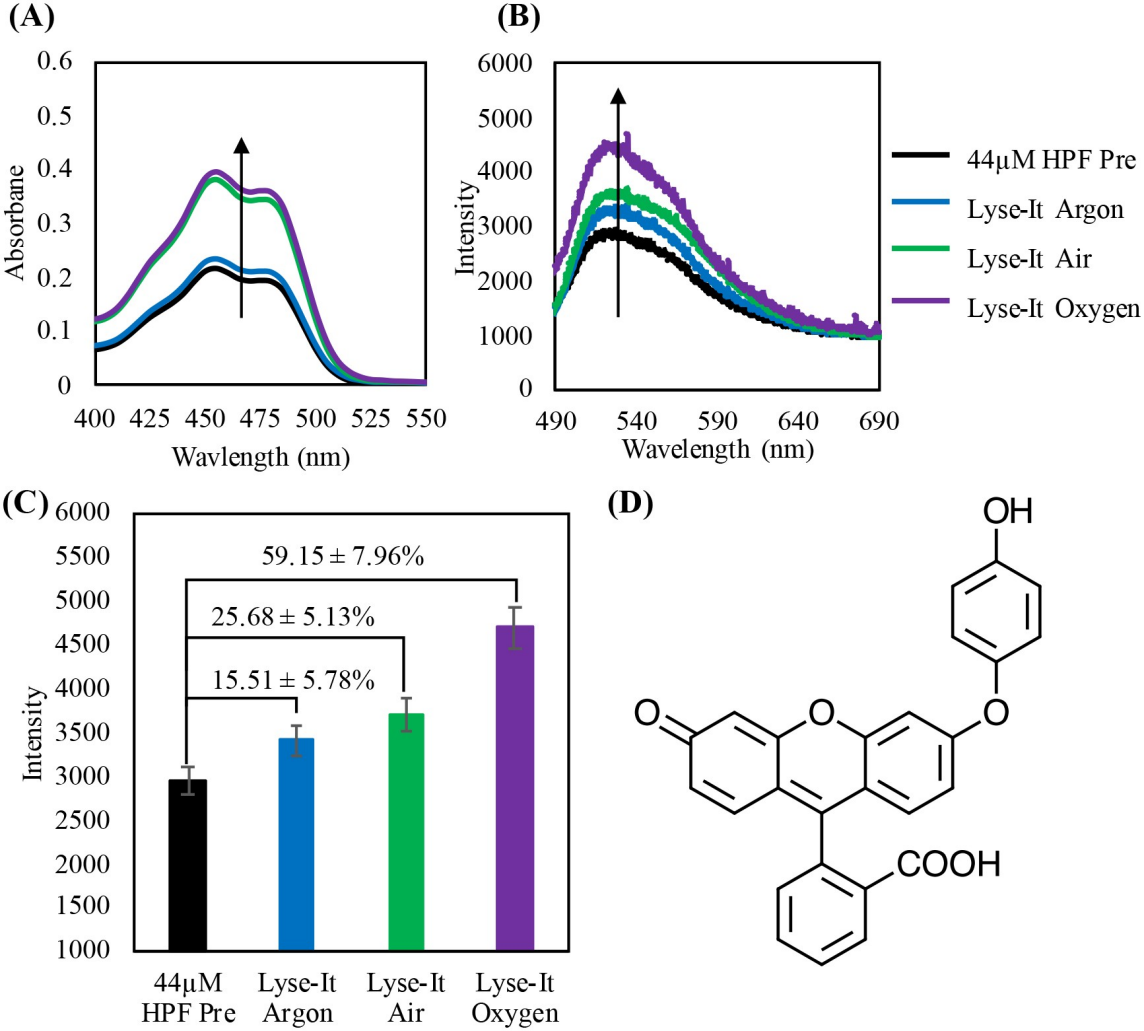
44 μM HPF purged then irradiated at 30% power, 60 seconds with Lyse-It **(A)** Absorption spectra of HPF pre and post microwaved irradiation, **(B)** Fluorescence spectra pre and post purging and microwave irradiation, **(C)** Fluorescence λ_max_ intensity with the percentage increase from *Pre* with respect to increasing oxygen concentration. **(D)** Hydroxyphenyl fluorescein unreacted probe. As oxygen content increases, the peak at approximately 450-nm in the absorbance spectra and 530-nm in the fluorescence spectra increases, indicating an increase in the detection of hydroxyl radicals.

Finally, 44 μM HPF was purged and then lysed at 50% power, 60 seconds with Lyse-It. Similar absorption, fluorescence spectra and percentage increases can be seen in Fig 8 under the same 5% λ_max_ error, as compared to the other ROS probes discussed. As oxygen concentration increased, there was an increase in the generation of hydroxyl radicals using Lyse-It, as specifically detected by HPF.

From all the fluorescent probe ROS data, a summary table was constructed (Table 1) showing the respective fluorescent intensity increases from the *Pre* probe values for DHE, HPF, and SOSG. It is clear and evident that as the oxygen concentration in the lysed sample increases, more reactive oxygen species in particular ^1^O_2_, ·OH, and O_2_^·-^, is detected by the specific fluorescent probes.[20, 23–26]

**Table 1:** Percentage increases in the *Pre* sample value for purged then microwave irradiated DHE, HPD, and SOSG with Lyse-It.

% Increase from Pre (0%)	DHE (O_2_^∙-^)	HPF (•OH)	SOSG (^1^O_2_)
**Lysed in Argon**	11.03 ± 0.01%	15.51 ± 5.78%	24.91 ± 5.10%
**Lysed in Air**	42.11 ± 0.01%	25.68 ± 5.13%	31.69 ± 6.58%
**Lysed in Oxygen**	43.37 ± 0.01%	59.15 ± 7.96%	45.20 ± 7.26%

### As microwave power increases, the generation of ^1^O_2_, ·OH, and O_2_^·-^ increases

After investigating the generation of ROS from increasing oxygenated environments and subsequent microwave irradiation at one microwave power and time setting (50% power, 60 seconds), it was interesting to see that if by additionally increasing the microwave power would also increase the generation of ROS. This experiment is particularly important as it answers the question as to whether microwave power is directly responsible for ROS generation. Thus, DHE, SOSG, and HPF, were microwave irradiated at 10, 30, and 50% power for 60 seconds and allowed to cool to room temperature prior to spectral analysis. Just like for increasing oxygenated environments above, the percent increase relative to the unreacted probe was calculated under the assumption of a 5% error for each of the λ_max_ fluorescent values. Relative error was then calculated from the high, original, and low λ_max_ for each sample (*increasing microwave power)* divided by the original *Pre* λ_max_.

**Fig 9:**
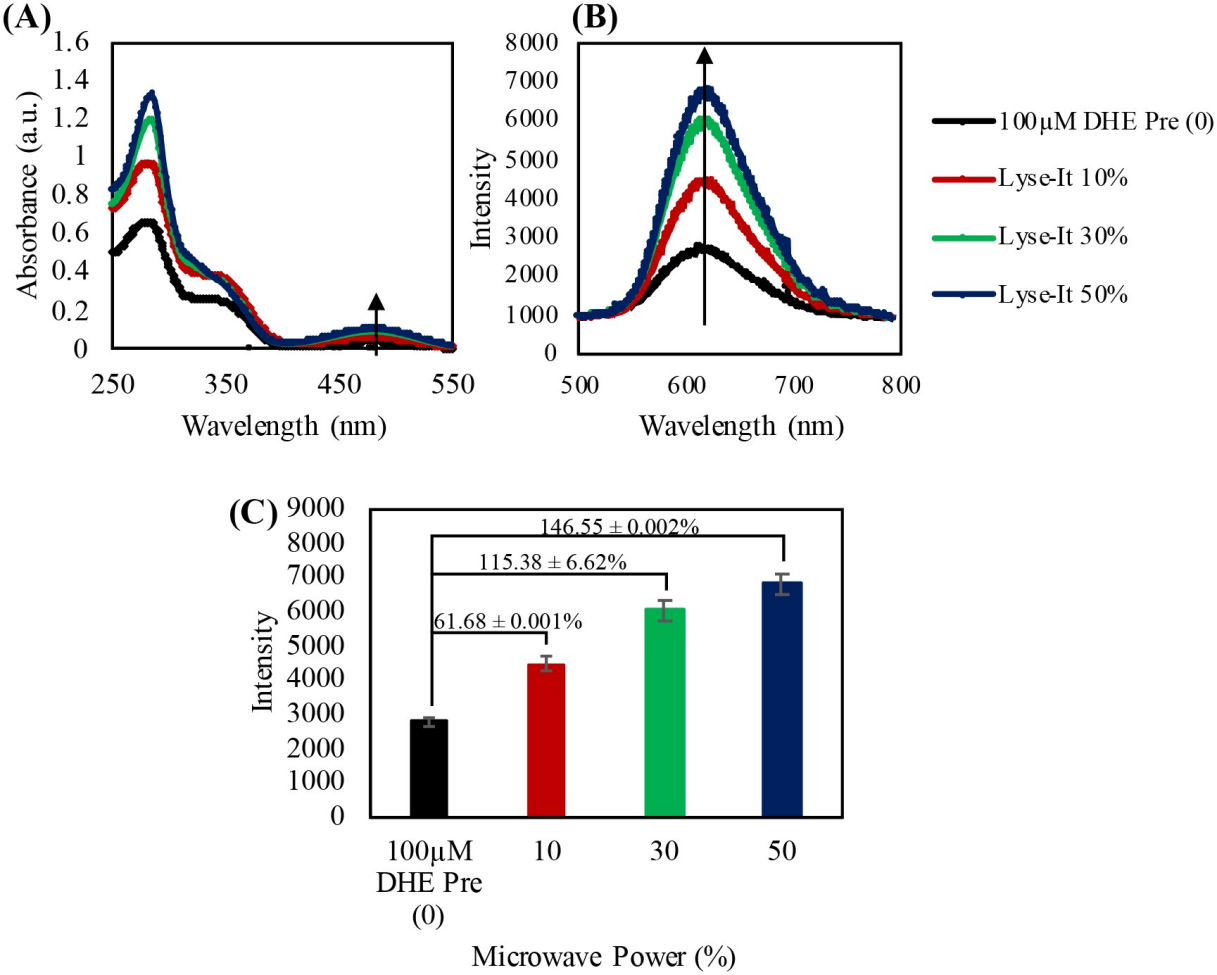
100 μM DHE microwave irradiated at 10%, 30%, and 50% power, 60 seconds with Lyse-It (A) absorption spectra of DHE pre and post microwaved irradiation, (B) fluorescence spectra pre and post purging and microwave irradiation, (C) fluorescence λ_max_ intensity with the percentage increase from *Pre* with respect to increasing microwave power. As microwave power increases, the peak at approximately 475-nm in the absorbance spectra and 605-nm in the fluorescence spectra increases indicating an increase in the detection of superoxide anion radical.

As shown in Fig 9A, as the microwave power increases, the generation of O_2_^·-^ increased as shown by the increase in the absorbance peak at 475 nm, and by the increase in fluorescence of the 2-hydroxyethidium product at 605-nm (Fig 9B). The percentage increase from the *Pre* value (initial control) was calculated for irradiation with Lyse-It and can be seen in Fig 9C. There was over a 140% increase in the detection of O_2_^·-^ when the probe was suspended in DI water and incubated in air and microwave irradiated at 50% power, 60 seconds. This increase was not as drastic from when the probe was purged in oxygen and then microwave irradiated with Lyse-It for 60 seconds at 50% power. We attribute this drastic fluorescence difference to oxygen being a quencher of fluorescence and thus when oxygen is in excess, the fluorescence of 2-hydroxyethidium will be diminished. This analysis of the decrease in the fluorescence with all the probes at 50% power, 60 seconds in oxygenated environments is consistent with molecular oxygen being a quencher of florescence. In agreement with DHE, as microwave power increased, ^1^O_2_ and ·OH increased as seen by the SOSG absorbance and fluorescence spectra (S5 Fig) and the HPF absorbance and fluorescence spectra (S6 Fig) respectively. The percent increases were calculated respectively for each of the probes and can be found in graph C of S5 and S6 Figs.

From this data, our findings strongly suggest that as the microwave power increases, solution ROS concentration dramatically increases as shown by the absorbance (ground state) changes of the probes and the respective product formation and subsequent increase in fluorescence of the resultant oxidized products. It is important to note that the oxidative products with the fluorescent probes are formed in the ground-state, as confirmed by the absorbance spectra. Therefore, samples were allowed to cool to room temperature before the fluorescent measurements were made, alleviating the need for any temperature corrections of the fluorescence emission data.

### More ROS are generated with the use of Lyse-It as compared to standard microwave irradiation (no Lyse-It)

**Fig 10:**
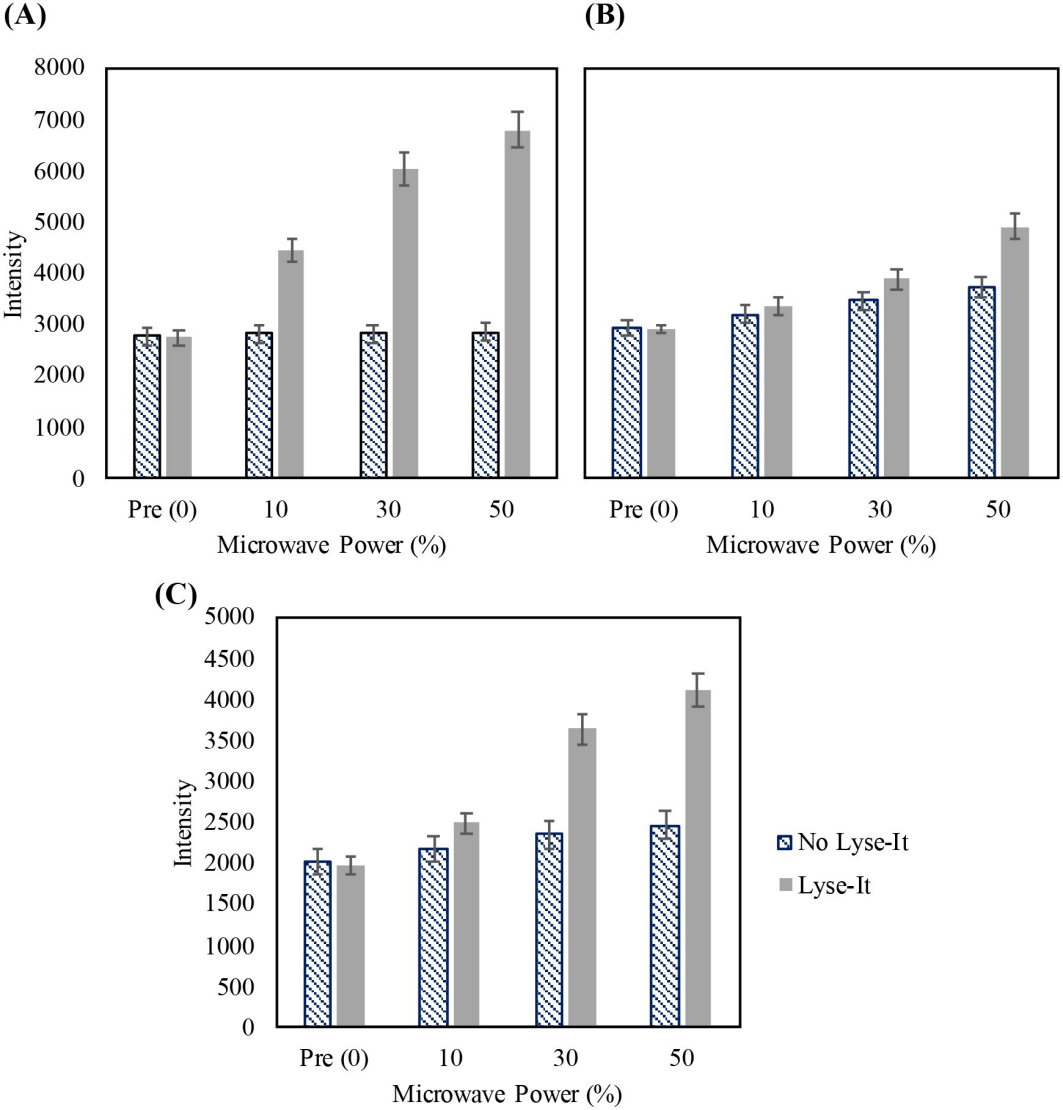
Detection of reactive oxygen species with fluorescent probes—A comparison of no Lyse-It to Lyse-It for 60 seconds. (A) 100 μM DHE (B) 22 μM SOSG (C) 44 μM HPF. There is more detection of reactive oxygen species with the use of Lyse-It as compared to no Lyse-It.

Following the investigation into the generation of increasing ROS with an increased microwave power, it was important to determine if using Lyse-It generates more ROS than simply using standard microwave irradiation. From the protein and DNA SDS PAGE and 2100 Bioanalyzer analysis, it was visually shown that when Lyse-It was used, more protein and DNA degradation did occur. Thus, it was expected that when using Lyse-It, there would be more generation of ROS to cut the proteins and DNA as compared to standard microwave irradiation. A comparison was subsequently performed with each of the three probes for O_2_^·-^, ^1^O_2_, and ·OH (Fig 10A, 10B and 10C **respectively**).

In all cases, when Lyse-It was used, there was a substantial increase in the formation of the probe/ROS species reaction product and subsequently an increase in the fluorescence. When only standard microwave irradiation was used, there was a negligible increase in the generation and subsequent detection of ROS as compared to irradiation with Lyse-It. Table 2 shows the respective percentage increases for each of the probes both *with* and *without* the use of Lyse-It with the assumption of a 5% error from the fluorescent intensity λ_max_. There are significant differences that clearly suggest that Lyse-It generates more ROS than standard microwave irradiation alone, ultimately degrading more proteins and DNA more effectively.

**Table 2:** Percentage increases from *Pre* for microwave irradiated DHE, HPF, and SOSG both with and without Lyse-It.

% Increase from Pre (0%)	DHE (O_2_^∙-^)	HPF (•OH)	SOSG (^1^O_2_)
**No Lyse-It 10%**	3.99 ± 3.59%	5.23 ± 4.55%	7.21 ± 5.36%
**Lyse-It 10%**	61.68 ± 0.001%	26.20 ± 0.006%	14.60 ± 2.87%
**No Lyse-It 30%**	3.96 ± 3.59%	10.51 ± 5.52%	17.97 ± 5.90%
**Lyse-It 30%**	115.38 ± 6.62%	84.47 ± 0.009%	33.04 ± 3.33%
**No Lyse-It 50%**	4.91 ± 3.62%	14.37 ± 5.72%	25.21 ± 6.26%
**Lyse-It 50%**	148.55 ± 0.002%	108.47 ± 0.01%	68.01 ± 4.20%

### Sample pH is not a factor in the increase in fluorescence for DHE, SOSG, or HPF and is thus not a factor for the increase in protein and DNA degradation with Lyse-It

**Fig 11:**
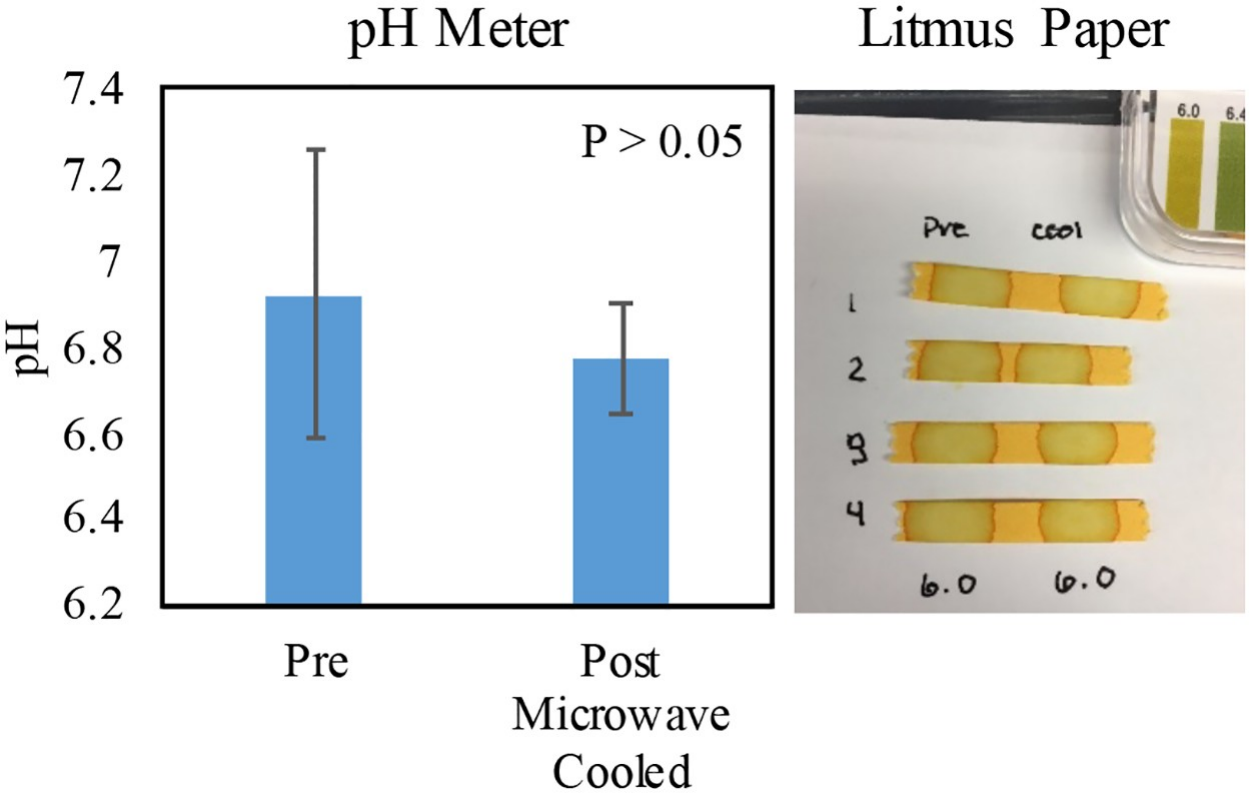
pH study of water *pre* and *post* microwave irradiation with Lyse-It at 50% power 60 seconds. There is no change in pH post microwave irradiation.

To confirm that the increase in the ROS probes was not due to a change in the sample pH, the pH of microwave irradiated water with Lyse-It was performed post microwave irradiation. Multiple irradiations of water were undertaken, and the pH of the water was tested with a pH meter and litmus pH paper. The pH of the DI water from the Institute of Marine and Environmental building water spickets was confirmed to be around pH 7 ± 0.4. The pH meter *Pre* microwave irradiated DI water was in agreement with the reported value. After microwave irradiation, the pH of water was obtained and the pH value was not statistically significant as compared to the *Pre* DI water pH value (Fig 11 **pH meter**). Visual analysis also confirmed that the pH did not change post microwave irradiation (Fig 11 **Litmus paper**).

### Oxidation of Fe^2+^ and Fe^3+^ occurs as sample oxygen content increases and microwave irradiation power and time increases with the use of Lyse-It

Considering that ROS are thought to be generated through microwave irradiation, we subsequently investigated potential oxidation effects of Fe^2+^ and Fe^3+^, i.e. can microwave irradiation also oxidize both Fe^2+^ and Fe^3+^ ions. This was deemed a particularly useful experiment as inorganic ions are not though to degrade when exposed to focused microwave fields, nor do they self-generate ROS, as speculated for some fluorescent probes.[23, 24]

**Fig 12:**
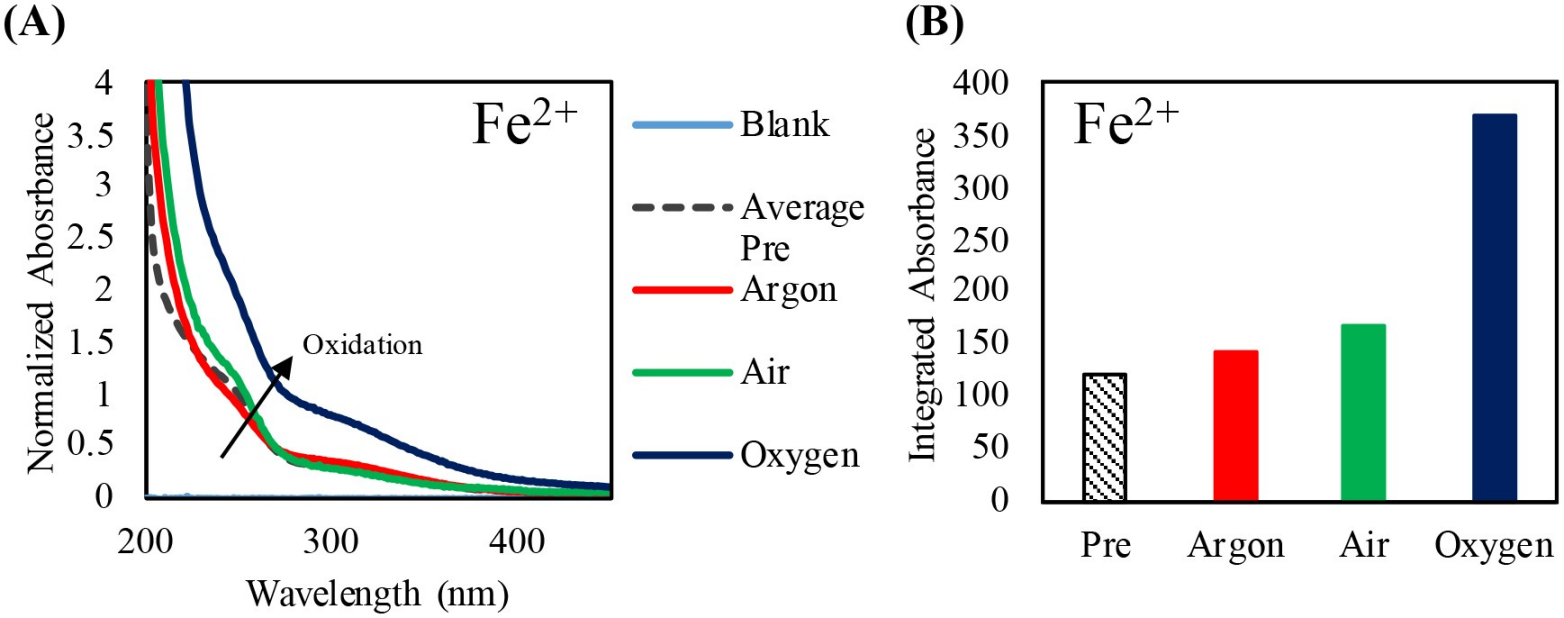
Purging study of 2.5 mM Fe (II) chloride and normalized at 250-nm post microwave irradiation with Lyse-It (30% power, 60 seconds) (A) and the integrated absorbance between 200 and 400-nm (B).

Iron samples were purged and then microwave irradiated at 30% power for 60 seconds. After irradiation, the samples were cooled to room temperature, absorbance spectra were obtained, and the *integrated* absorbance was calculated between 200 and 400-nm (Fig 12). Consistent with our studies of Fe^2+^ oxidized by 15.75% hydrogen peroxide (S7 Fig), Fe^2+^ likewise became oxidized to Fe^3+^ with increasing oxygen content. This aids in confirming that oxidation is present with increasing oxygen content and subsequent generation of ROS, as ROS are known to be powerful oxidizers.

**Fig 13:**
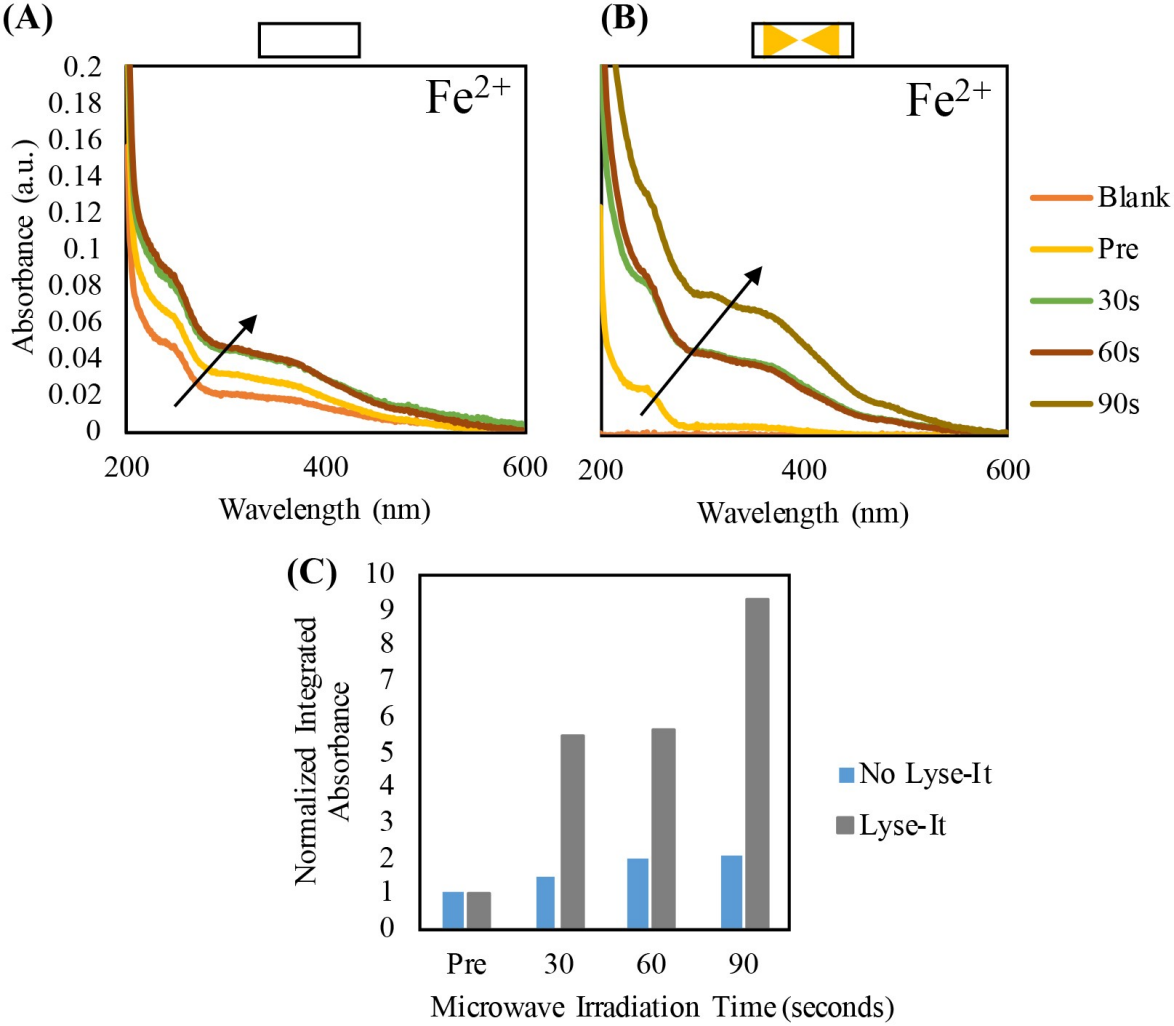
Absorbance spectra and the normalized integrated absorbance for increasing irradiation time of a solution of 2.5 mM Iron (II) chloride without Lyse-It **(A)** The irradiation time increases with 30% power with Lyse-It **(B)**. A comparison of the normalized integrated absorbance between no Lyse-It and Lyse-It **(C)**. Normalized integrated absorbance was taken between 200-600-nm.

One of the most pivotal experiments to confirm an increase in metal oxidation through microwave irradiation was to compare standard microwave irradiation to irradiation using Lyse-It. A 2.5 mM Iron (II) chloride solution was used and 30% microwave power (270W) was kept constant while the irradiation time was increased. Samples were subsequently irradiated both *with* or *without* Lyse-It for 30, 60, or 90 seconds (i.e. 8.1, 16.2, and 24.3 kJ total energy respectively). Absorbance spectra were obtained after the samples were cooled and it was found that by using Lyse-It, there was a substantial increase in oxidation as compared to just using standard microwave irradiation alone. These differences can be readily seen in the *with* and *without* Lyse-It absorbance spectra, as well as by considering the integrated absorbance between 200 and 600-nm (Fig 13).

**Fig 14:**
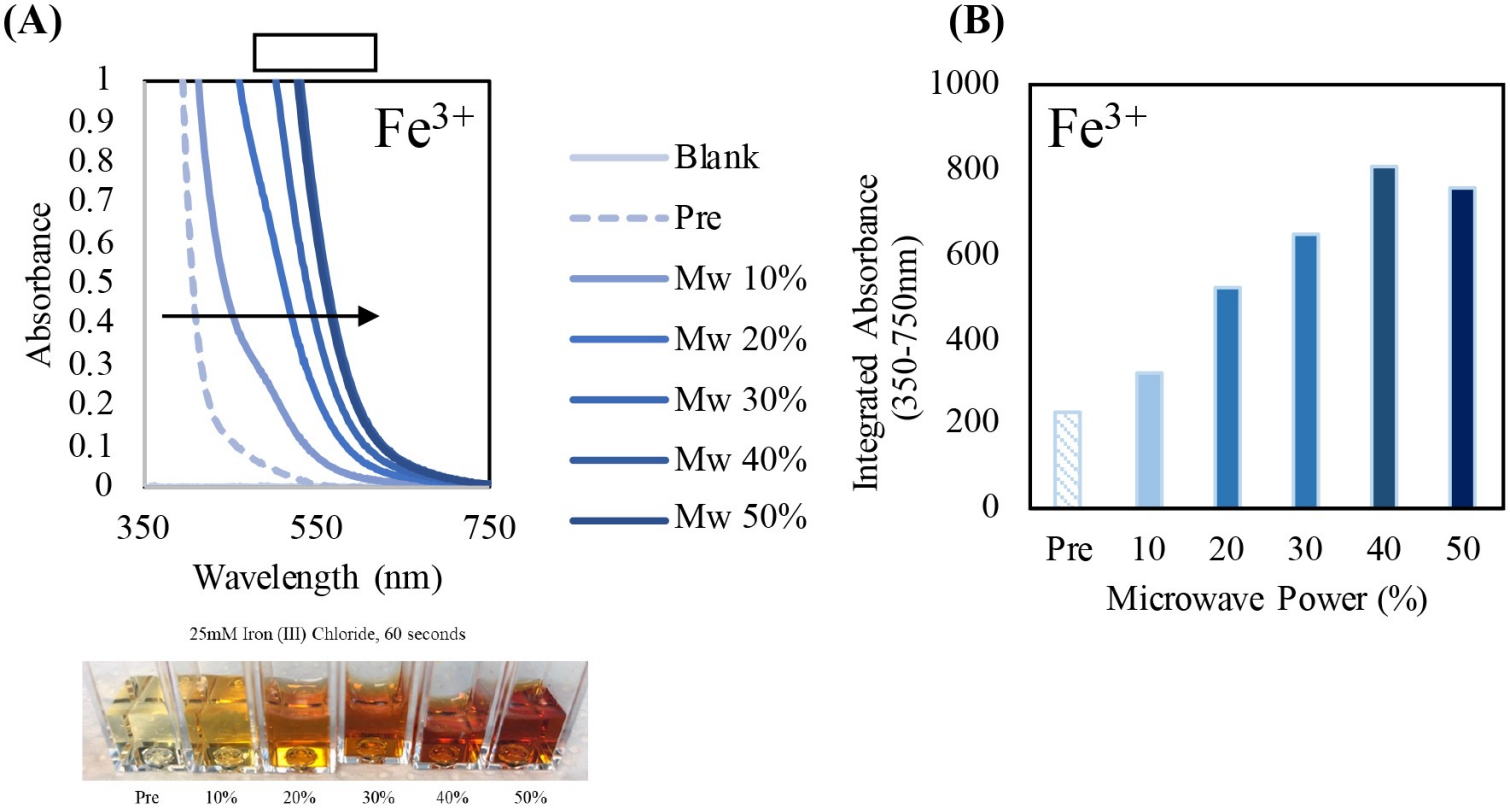
Absorbance spectra of 25 mM Iron (III) chloride and visual representation of the color change as a function of increased microwave power (Mw) (A) and the integrated absorbance between 350nm-750-nm (B) post microwave irradiation without Lyse-It for 60 seconds. As microwave power increases, Fe^3+^ gets progressively oxidized to iron oxide.

Additionally, a much higher concentration of Iron (III) chloride (25 mM) was also used to visually see the oxidation as microwave power increases and irradiation time was held constant. Visually it is seen that as microwave power is increased then more oxidation occurs of Fe^3+^ to iron oxide. There was an increase in the deep red solution color with increasing microwave power as well as the respective bathochromic shift in the absorbance spectra (Fig 14A) characteristic of the color of the iron oxide. The integrated absorbance was also calculated to demonstrate that as microwave power increases, a deep red color appears, and the absorbance the bathochromic shift becomes much greater (Fig 14B).

## Conclusions and discussion

Lyse-It is a rapid, tunable sample preparation platform for cellular lysis, intracellular component release, DNA/RNA and protein/enzyme fragmentation and inactivation.[6, 14, 17] It has been shown that bacterial cells can be lysed and at low microwave power and time, intracellular components are released without extensive degradation. However, as microwave power and/or time and thus overall microwave cavity energy increases, DNA, RNA, proteins, and enzymes become increasingly fragmented and/or inactivated.[6, 13, 14] Further analysis into DNA fragmentation and protein degradation from gel electrophoresis and 2100 Bioanalyzer studies subsequently led to the investigation of a non-thermal component connected with the use of Lyse-It, ultimately showing significantly increased levels of ROS generation.

Purging studies that increased the overall oxygen content in the sample were performed on ground spinach, ground beef, and various bacteria to elucidate the effect of oxygen content on protein and DNA fragmentation respectively. It was shown, that as oxygen content increases while microwave power and time were held constant, there was an increase in the degradation of proteins, irrespective of the sample temperature. Temperature had minimal effect on the degree of protein or DNA degradation as the microwave irradiation power and time increased. This was particularly evident for DNA fragmentation. At low microwave powers and short irradiation times, there are substantially more small base pair fragments in the presence of oxygen as compared to samples purged with argon (~0% O_2_) or equilibrated in air (~16% O_2_) when both microwave power and time were held constant. As microwave power and time is increased (30% power, 30 seconds to 50% power, 60 seconds), the amount of fragmentation in the oxygenated environment significantly increases as seen by the many orders of magnitude change in total concentration of DNA base pairs (pg/μL) for both Gram-positive and–negative bacteria. This data showed that there is compelling evidence for a non-thermal mechanism associated with increased protein and DNA fragmentation using Lyse-It. Thus we subsequently investigated the generation of *specific* reactive oxygen species with Lyse-It as these species, in particular singlet oxygen (^1^O_2_), hydroxyl radicals (•OH), and superoxide anion radicals (O_2_^•-^) are known to be associated with intracellular component damage.[27–30] Commercially available off/on fluorescent-based probes were used to detect these radical species during microwave irradiation both *with* and *without* Lyse-It. In more oxygenated environments, more ROS were detected as shown by an increase in fluorescence for all of the ROS specific probes. Additionally, when comparing both standard microwave irradiation (i.e. no Lyse-It) to Lyse-It, there was substantially more ROS generated using Lyse-It than standard microwave irradiation. This was astounding as this increase in the detection of ROS supports all previous work utilizing Lyse-It, i.e. that it is not just a temperature dependent biomolecule degradation mechanism, but moreover, fragmentation is also dependent on a non-thermal ROS component.

Further investigation of the oxidative effects of Lyse-It included investigating the oxidative effects on metal ions, in particular Fe^2+^ and Fe^3+^, as metal ions are not thought to degrade when exposed to microwaves. Similarly, to purging protein and DNA, metal ions were purged with increasing oxygen content and subsequently microwave irradiated. It was found that as oxygen content increased, there was an increase in the degree of metal ion oxidation. Additionally, metal ion oxidation was compared both *with* and *without* Lyse-It. Lyse-It oxidized more metal ions at an increased rate than standard microwave irradiation alone.

In summary, this paper represents strong evidence of a secondary biomolecule fragmentation mechanism underpinning Lyse-It, which cannot simply be attributed to temperature (i.e. the generation of reactive oxygen species). Specific fluorescent probes for singlet oxygen, hydroxyl radicals, and superoxide anion radicals confirm their increased concentration both in elevated oxygen environments, as well as when using Lyse-It versus no Lyse-It control samples.

## Supporting information

S1 Fig A 1 mL Lyse-It slide (A) and the Lyse-It purging system experimental setup (B). (TIF)

S2 Fig SDS PAGE of ground spinach conventional heating, standard microwave lysing, and lysing with Lyse-It **(A)**. 3D images of the boxed regions of spinach conventionally heating from 40–90°C for 1 minute **(B)**. Conventional heating (left) spinach lysed without Lyse-It (middle) and with Lyse-It (right). More protein was extracted and subsequently degraded with Lyse-It as a function of increasing microwave power. L: kDa ladder, Lanes 1–6: 40–90°C, Lanes 7–12: standard microwave irradiation (no Lyse-It) 10–60% power, 60 seconds, Lanes 13–19: microwaving with Lyse-It 10–60% power, 60 seconds.
(TIF)

S3 Fig SDS PAGE of purged ground spinach with and without Lyse-It (30% power, 60 seconds). Protein extraction is seen with the Pre (P) ground food samples. Protein degradation is then seen as the oxygen content increases (Argon to Air to Oxygenated purged samples).
(TIF)

S4 Fig 2100 high sensitivity DNA Bioanalyzer of *L*. *monocytogenes*. Bacteria were purged for 15 minutes and then lysed with Lyse-It for 30 seconds at 30% power **(A and B)** and 60 seconds at 50% power **(C and D)**. **A and C)** Total concentrations (pg/μL) below the reported base pairs. **B and D)** Concentrations (pg/μL) between base pair ranges.
(TIF)

S5 Fig 22 μM SOSG purged then irradiated at 50% power, 60 seconds with Lyse-It (A) absorption spectra of SOSG pre and post microwaved irradiation, (B) fluorescence spectra (ex: 473 nm) pre and post purging and microwave irradiation, (C) fluorescence λ_max_ intensity with the percentage increase from *Pre* with respect to increasing microwave power. As microwave power increases, the peak at approximately 475-nm in the absorbance spectra and 525-nm in the fluorescence spectra increases indicating an increase in the detection of singlet oxygen.
(TIF)

S6 Fig 44 μM HPF purged then irradiated at 50% power, 60 seconds with Lyse-It (A) absorption spectra of HPF pre and post microwaved irradiation, (B) fluorescence spectra (ex: 473 nm) pre and post purging and microwave irradiation, (C) fluorescence λ_max_ intensity with the percentage increase from *Pre* with respect to increasing microwave power. As microwave power increases, the peak at approximately 475-nm in the absorbance spectra and 525-nm in the fluorescence spectra increases indicating an increase in the detection of hydroxyl radicals.
(TIF)

S7 Fig 2.5 mM Iron (II) chloride oxidized with 15.75% hydrogen peroxide additions. (TIF)
