# Supplementary figures and images for: Elucidation of a non-thermal mechanism for DNA/RNA fragmentation and protein degradation when using Lyse-It
Source: PLoS One. 2019 Dec 2;14(12):e0225475. doi: 10.1371/journal.pone.0225475 (PMC6886747; doi:10.1371/journal.pone.0225475)

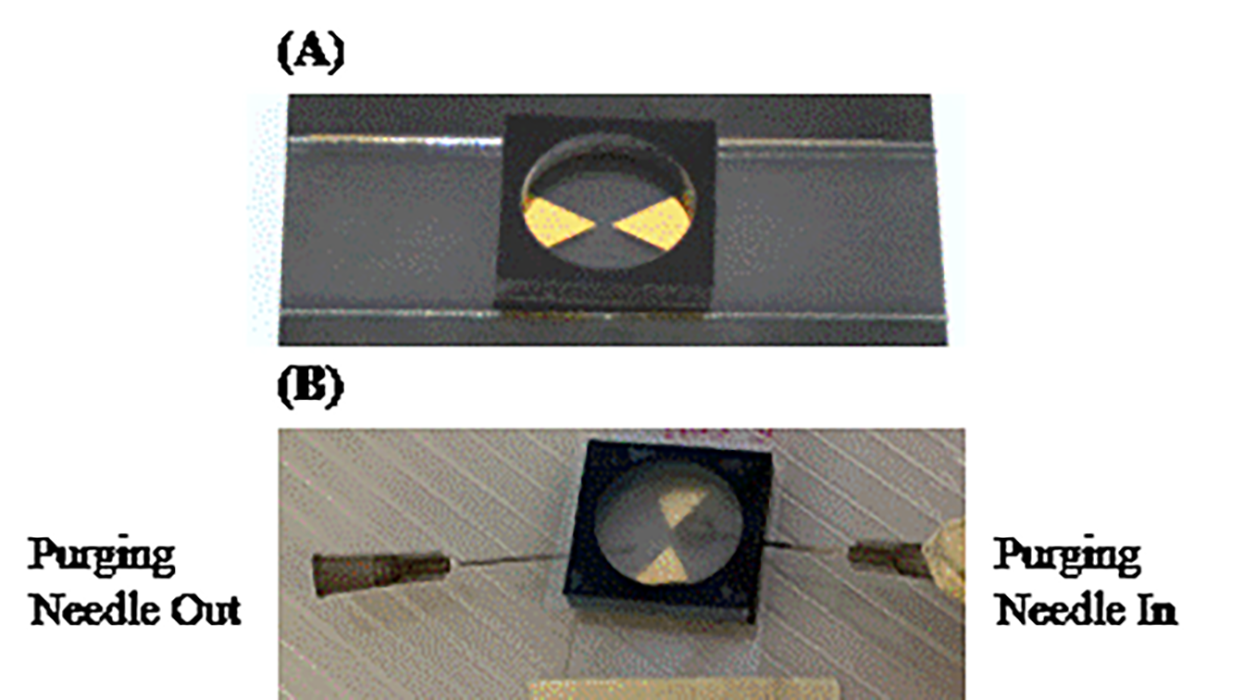

Supplement: S1 Fig — (TIF) [file pone.0225475.s001.tif]

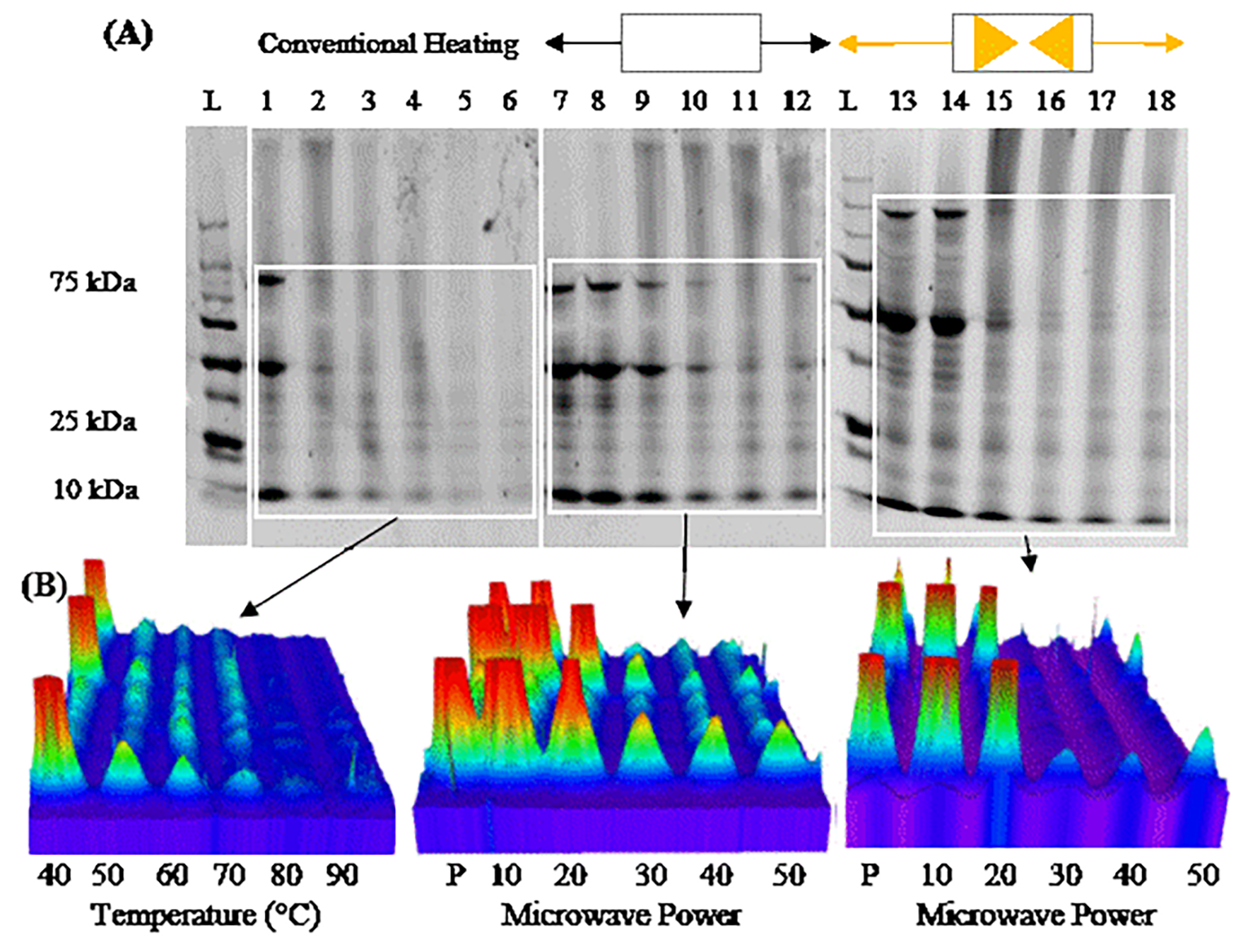

Supplement: S2 Fig — SDS PAGE of ground spinach conventional heating, standard microwave lysing, and lysing with Lyse-It (A). 3D images of the boxed regions of spinach conventionally heating from 40–90°C for 1 minute (B). Conventional heating (left) spinach lysed without Lyse-It (middle) and with Lyse-It (right). More protein was extracted and subsequently degraded with Lyse-It as a function of increasing microwave power. L: kDa ladder, Lanes 1–6: 40–90°C, Lanes 7–12: standard microwave irradiation (no Lyse-It) 10–60% power, 60 seconds, Lanes 13–19: microwaving with Lyse-It 10–60% power, 60 seconds. (TIF) [file pone.0225475.s002.tif]

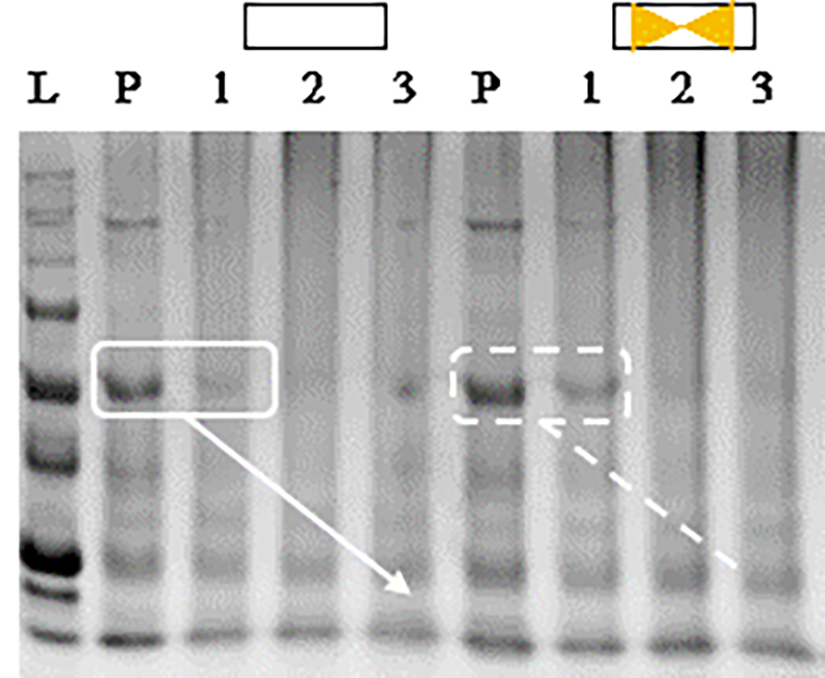

Supplement: S3 Fig — Protein extraction is seen with the Pre (P) ground food samples. Protein degradation is then seen as the oxygen content increases (Argon to Air to Oxygenated purged samples). (TIF) [file pone.0225475.s003.tif]

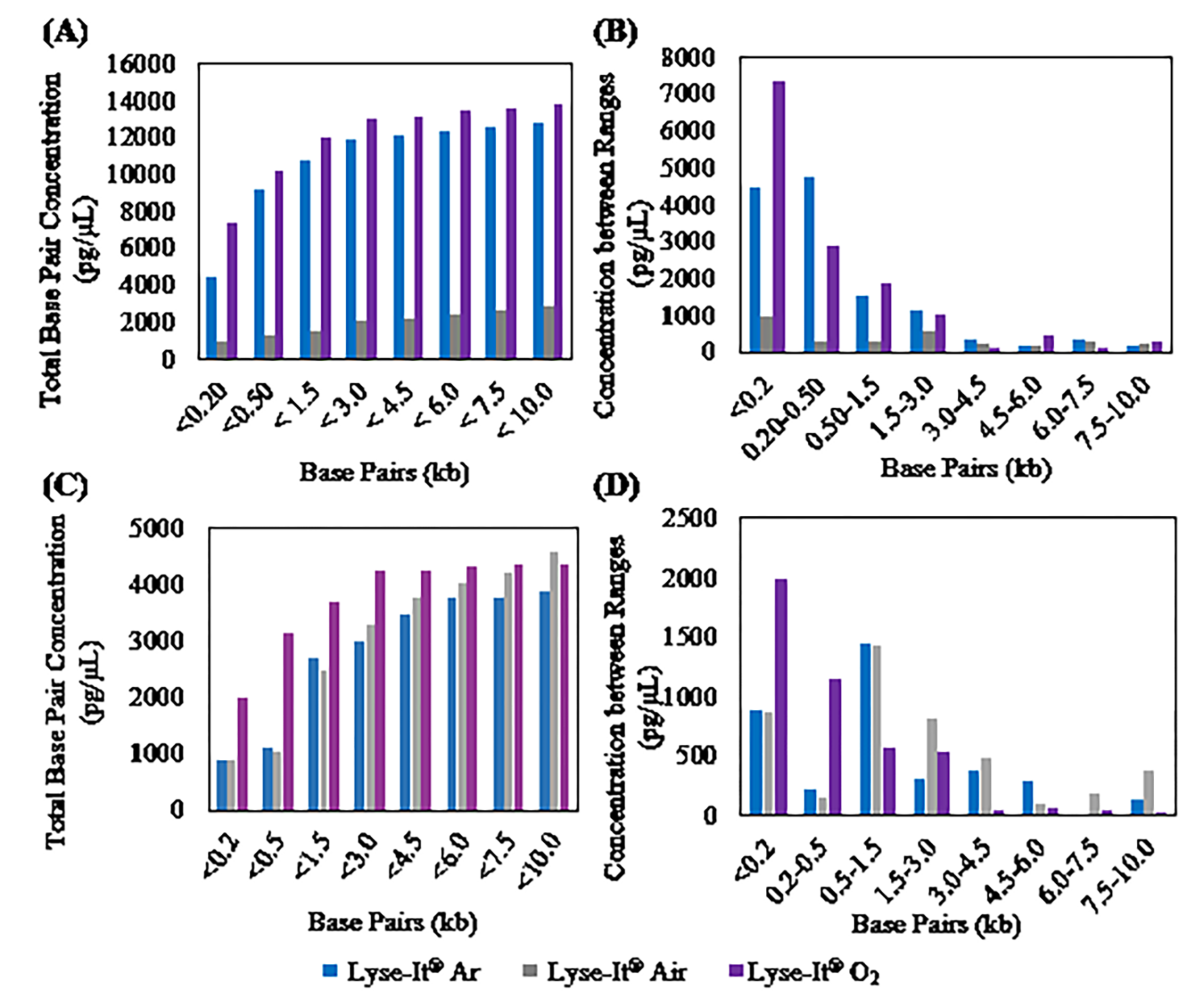

Supplement: S4 Fig — Bacteria were purged for 15 minutes and then lysed with Lyse-It for 30 seconds at 30% power (A and B) and 60 seconds at 50% power (C and D). A and C) Total concentrations (pg/μL) below the reported base pairs. B and D) Concentrations (pg/μL) between base pair ranges. (TIF) [file pone.0225475.s004.tif]

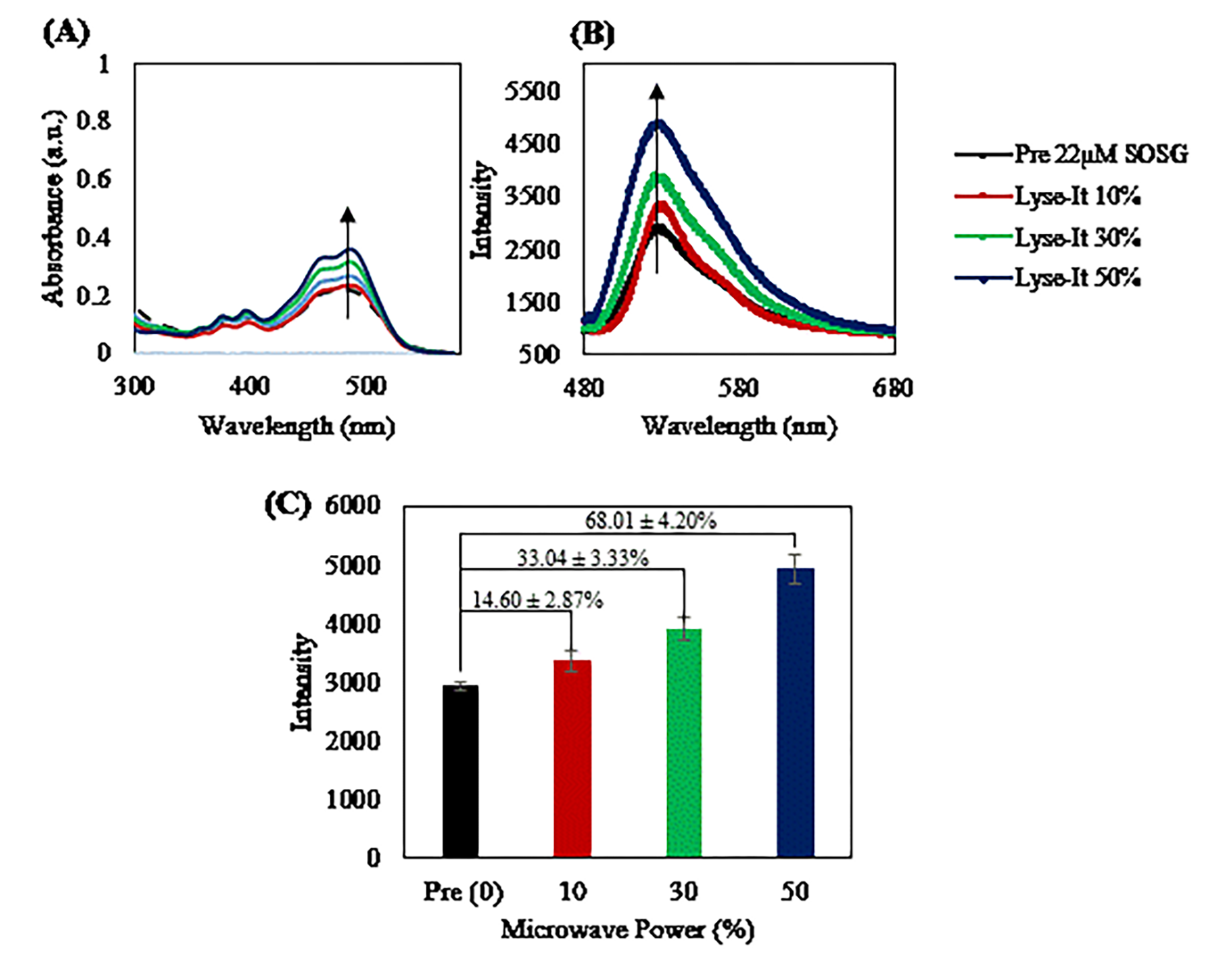

Supplement: S5 Fig — As microwave power increases, the peak at approximately 475-nm in the absorbance spectra and 525-nm in the fluorescence spectra increases indicating an increase in the detection of singlet oxygen. (TIF) [file pone.0225475.s005.tif]

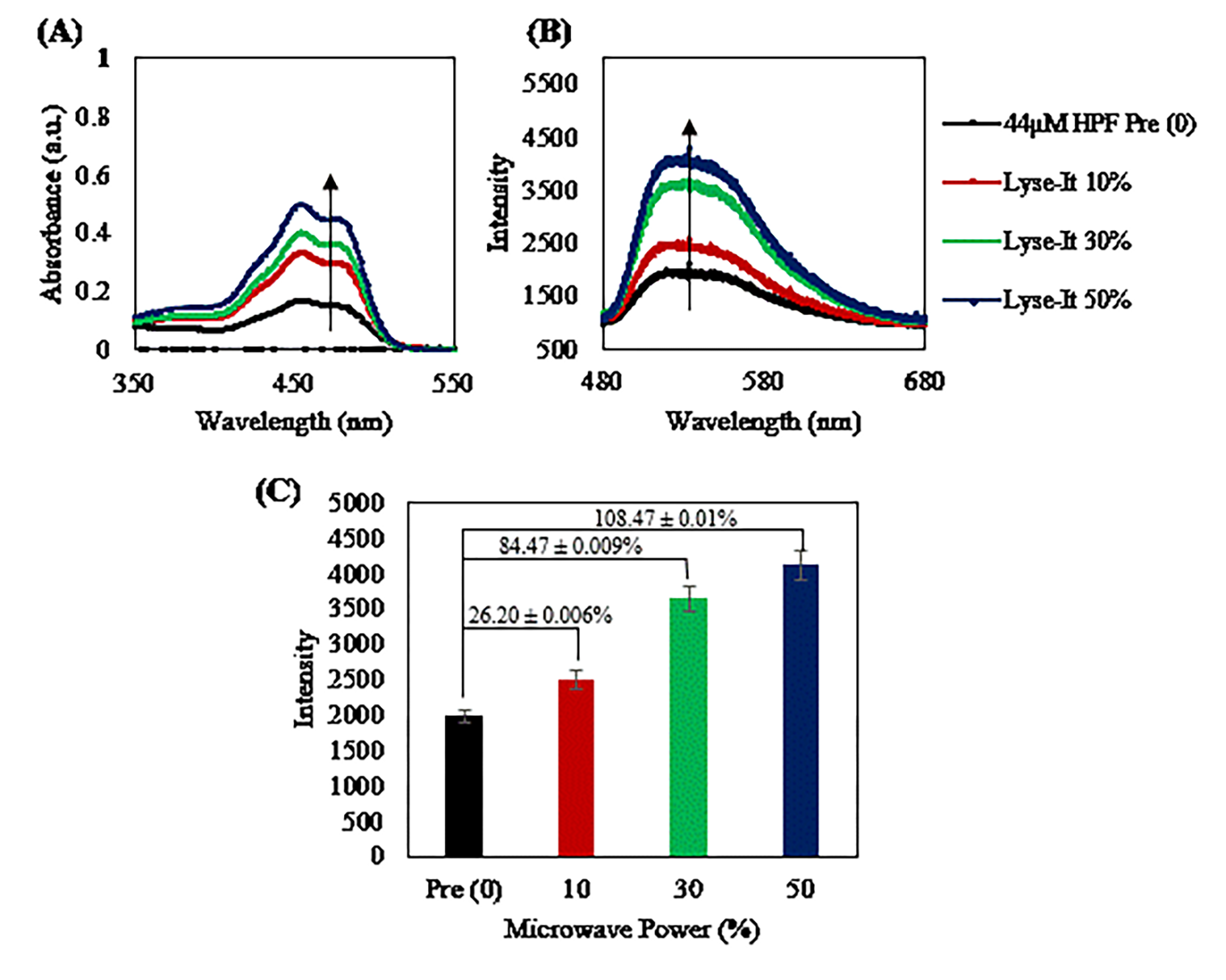

Supplement: S6 Fig — As microwave power increases, the peak at approximately 475-nm in the absorbance spectra and 525-nm in the fluorescence spectra increases indicating an increase in the detection of hydroxyl radicals. (TIF) [file pone.0225475.s006.tif]

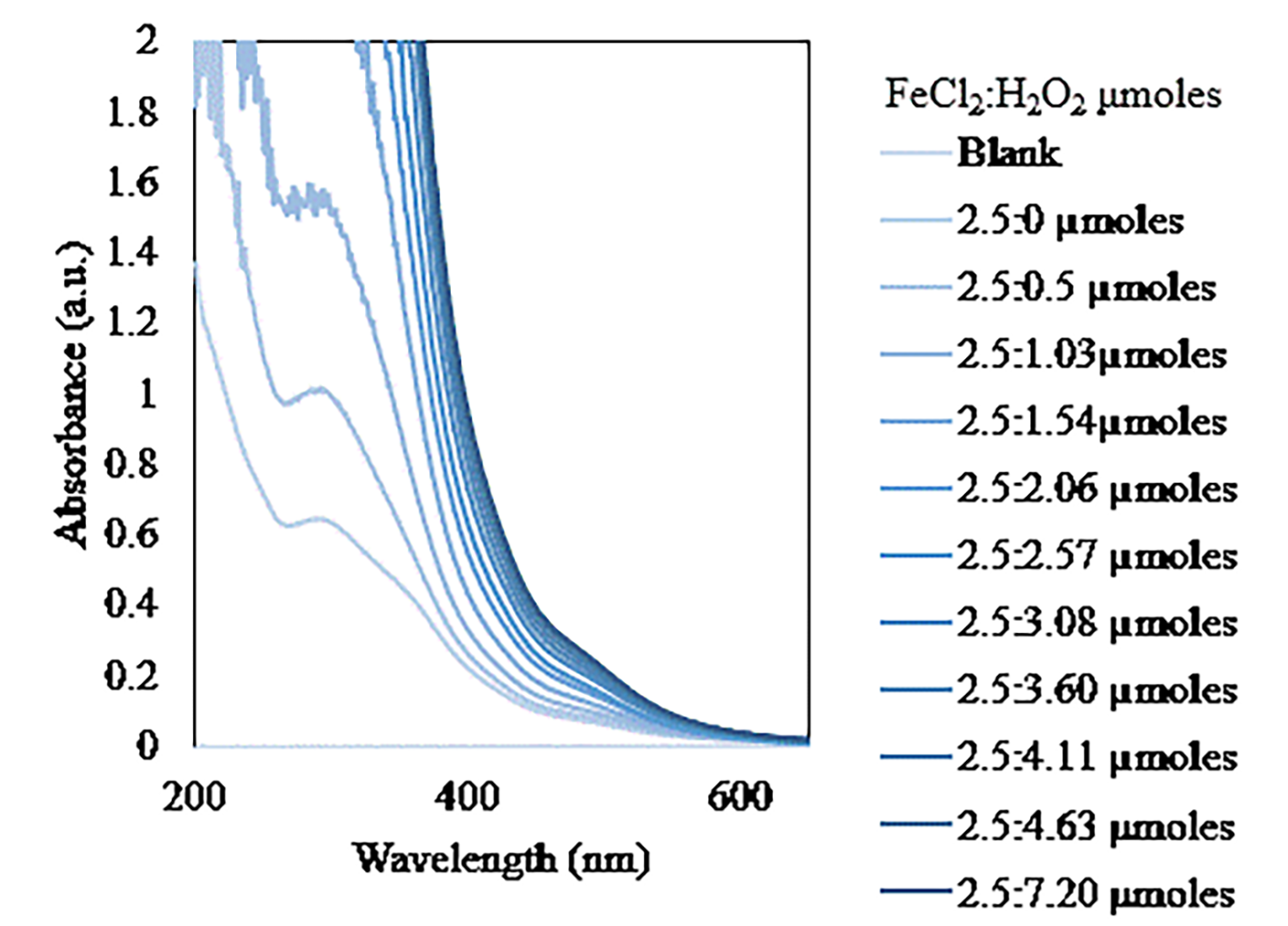

Supplement: S7 Fig — (TIF) [file pone.0225475.s007.tif]
